# A rational blueprint for the design of chemically-controlled protein switches

**DOI:** 10.1038/s41467-021-25735-9

**Published:** 2021-10-01

**Authors:** Sailan Shui, Pablo Gainza, Leo Scheller, Che Yang, Yoichi Kurumida, Stéphane Rosset, Sandrine Georgeon, Raphaël B. Di Roberto, Rocío Castellanos-Rueda, Sai T. Reddy, Bruno E. Correia

**Affiliations:** 1 Laboratory of Protein Design and Immunoengineering (LPDI) - STI - EPFL, Lausanne, Switzerland; 2 Swiss Institute of Bioinformatics (SIB), Lausanne, CH-1015 Switzerland; 3 Department of Life Science, School and Graduate School of Bioscience and Biotechnology, Tokyo Institute of Technology, Meguro-ku, Tokyo, 152-8550 Japan; 4 Department of Biosystems Science and Engineering, ETH Zürich, 4058 Basel, Switzerland

**Keywords:** Synthetic biology, Protein design

## Abstract

Small-molecule responsive protein switches are crucial components to control synthetic cellular activities. However, the repertoire of small-molecule protein switches is insufficient for many applications, including those in the translational spaces, where properties such as safety, immunogenicity, drug half-life, and drug side-effects are critical. Here, we present a computational protein design strategy to repurpose drug-inhibited protein-protein interactions as OFF- and ON-switches. The designed binders and drug-receptors form chemically-disruptable heterodimers (CDH) which dissociate in the presence of small molecules. To design ON-switches, we converted the CDHs into a multi-domain architecture which we refer to as *activation by inhibitor release* switches (AIR) that incorporate a rationally designed drug-insensitive receptor protein. CDHs and AIRs showed excellent performance as drug responsive switches to control combinations of synthetic circuits in mammalian cells. This approach effectively expands the chemical space and logic responses in living cells and provides a blueprint to develop new ON- and OFF-switches.

## Abstract (web summary)

Small-molecule responsive protein switches are crucial components to control synthetic cellular activities. Here, we present a computational protein design strategy to repurpose drug-inhibited protein-protein interactions into OFF- and ON-switches active in cells.

## Introduction

Synthetic biology has enabled important developments on the understanding of fundamental aspects in biology as well as in next-generation cell-based therapies^1–3^. In synthetic biology, many strategies have been pursued to control timing, localization, specificity and strength of transgene expression or signaling events by equipping cells with sophisticated genetic circuits governed by small-molecule controlled protein switches. Typically, protein switches are triggered by a small molecule to control the assembly or disassembly of two protein subunits^1^. One of the most widespread switch systems is the rapamycin-controlled chemically induced dimers (CIDs), FKBP:FRB which has been used in a wide variety of applications, including to control chimeric antigen receptor T (CAR-T) cell activities as safety switches^4^.

However, the large majority of chemically-controlled protein switches have limitations, particularly in translational applications, due to drug toxicity/side effects^5,6^ and unfavorable pharmacokinetics^7^, or concerns that non-human protein components could raise an immunogenic response^8–10^. Thus, an important requirement to enhance the breadth and scope of synthetic biology applications is to expand the universe of protein-based switches and consequently of the chemical space used to control engineered cellular activities.

Beyond naturally-sourced protein switches^1^, several methods have recently been proposed to expand the panel of available protein switches. Specifically for CIDs, Hill and colleagues used an in vitro evolution-based approach to engineer antibodies that engage with Bcl-XL only in the presence of a small-molecule drug^11^, and showed that these switches were active in cellular applications. Foight and colleagues used libraries of computationally designed mutants of a previously reported de novo protein scaffold to interact with a viral protease only in its drug-bound state^12^. These were also shown to regulate cellular activities in vivo, but the crystal structures of the designs evidenced substantial differences to the predicted binding modes. Remarkable computational design work was performed by Glasgow and colleagues, where a CID was rationally designed by transplanting the binding sites of a ligand to an existing protein dimer^13^. However, the precise design of key interaction residues to mediate small molecule interactions and control CIDs remain an extremely challenging computational design problem.

All these approaches focus on chemically induced dimerization systems^1,11–13^, yet chemical disruption systems also have important applications in synthetic biology and remain much less explored^14,15^. Therefore, we devised a strategy to design chemically-controlled switches by repurposing protein components and small molecules involved in the inhibition of protein-protein interactions (PPI)^16^. Multiple PPI inhibitors have been in clinical development over the past few years and some are approved for clinical use^16,17^, which make them attractive molecules for synthetic biology applications.

Previously, we reported the design of chemically disruptable heterodimers (CDH) from known protein: peptide-motif complexes, by transferring the peptide motif from the disordered binding partner to globular proteins and using the known PPI inhibitor as a chemical disruptor^18^. Specifically, our CDH was based on the Bcl-XL:BIM-BH3 complex, where the BIM-BH3 interaction motif was transplanted to the Lead Design 3 (LD3) using computational design, resulting in a Bcl-XL binder with 1000-fold times higher affinity than the wildtype disordered BIM-BH3 peptide-motif. Biochemically, we showed that Bcl-XL:LD3 complex (CDH-1) is disrupted by the drug A-1155463 (Drug-1)^19^, and can be used as an OFF switch for CAR-T cell activity in a dose-dependent, dynamic, and reversible manner in vivo^18^.

**Fig. 1:**
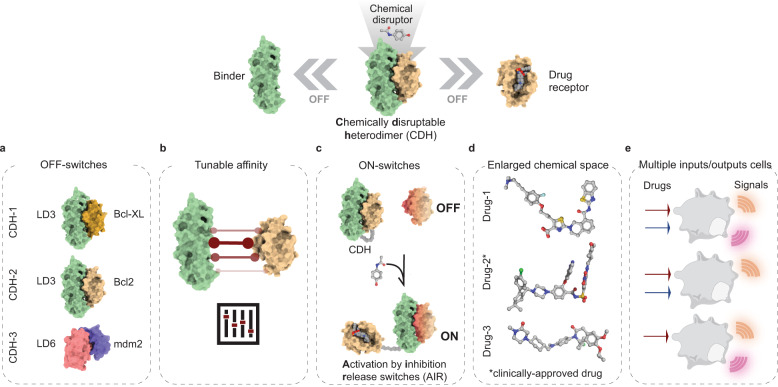
Overview of structure-based design strategies employed to create novel small-molecule responsive switches. These design strategies include: (I) binding site transplantation to create new CDHs (**a**) that can be used to control cellular activities; (II) Interface mutations for affinity tuning (**b**) to alter drug concentrations that interfere with assembly of the complexes; (III) Multistate design to create drug insensitive receptors that are embedded in multi-domain architectures that dimerize upon drug exposure (**c**). This protein switch toolbox enlarges the chemical space (**d**) and can be used for the engineering of cells which sense several chemical cues and output a variety of signals (**e**).

Here, we expand our computationally-driven approach to design novel chemically controlled protein switches using distinct small-molecule drugs. First, we designed two new CDHs: CDH-2 based on the Bcl2:LD3 protein complex disrupted by the clinically-approved drug Venetoclax (Drug-2)^20,21^; CDH-3 based mdm2:p53 complex disrupted by NVP-CGM097 (Drug-3)^22^ (Fig. 1a). The three CDHs (CDH-1-3) were used to regulate cellular responses, which included transcriptional gene expression^23^ and synthetic cell surface receptor signaling based on the GEMS platform^24^. In order to investigate the impact of biochemical parameters on drug disruptor dosage, we designed a suite of CDHs with tailored affinity ranging from low picomolar to mid nanomolar and tested their effect in regulating cellular processes (Fig. 1b). Next, we devised a novel strategy to create ON switches (i.e. protein pairs where a small-molecule drug promotes heterodimerization) by repurposing the CDHs. These new switches, referred to as *activation by inhibitor release switch* (AIR), have a “split” architecture with two protein chains where one of the chains contains the two domains of a CDH genetically fused, and the second chain contains a rationally-designed drug insensitive receptor that retains the ability to bind to the computationally designed binder (e.g. LD3). The drug disruptor releases the designed binder from the fused CDH exposing the interaction site of the binder and consequently enabling the dimerization with the drug insensitive receptor (Fig. 1c). Notably, as the array of the clinically-validated PPI inhibitors grows^16^, our AIR strategy could be used to accelerate the development of ON-switches to control activities in engineered cells, which remains an important need in synthetic biology (Fig. 1d). Finally, we engineered mammalian cells equipped with multi-input/output control modes which showcased the broad applicability of the designed protein switches into more sophisticated control modes broadening the potential scope of applications (Fig. 1e).

Altogether, we provide a rational blueprint that leverages structure-based and computational design approaches to design novel small-molecule switches.

## Results

### Design of CDHs controlled by clinically relevant drugs

**Fig. 2:**
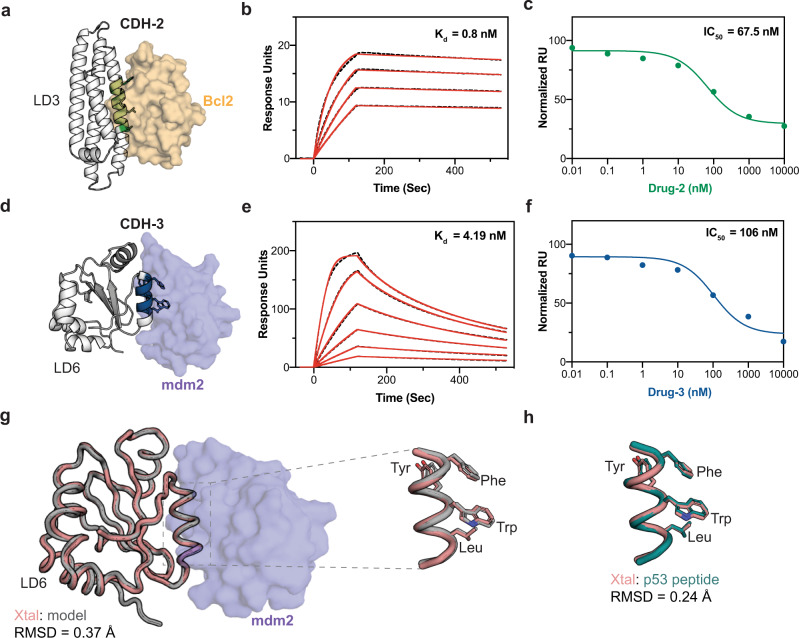
Biochemical and structural characterization of novel CDHs. **a** Structural representation of CDH-2 composed of Bcl2 (beige surface) and LD3 (white cartoon) with the interfacial segment colored in green, the hotspot residues transplanted from the BH3 motif are shown in sticks. **b** SPR measurements of CDH-2 binding affinity. The dissociation constant determined for the interaction of Bcl2 with LD3 is 796 pM. **c** SPR drug competition assay determined the apparent IC_50_ of CDH-2 with Drug-2 around 67.5 nM. **d** Structural representation of CDH-3 composed of the mdm2 (purple surface):LD6 (white cartoon) complex with the interfacial segment colored in blue, the hotspot residues transplanted from the p53 are motif shown in sticks. **e** SPR measurements of CDH-3 binding affinity. The dissociation constant determined for the interaction of mdm2 with LD6 is 4.19 nM. **f** SPR drug competition assay determined the apparent IC_50_ of CDH-3 with Drug-3 around 106 nM. **g** The crystal structure of the CDH-3 consisting of the complex LD6 (pink tube) with mdm2 (purple surface) was in close agreement with the computational model of LD6 (gray tube) with complex with mdm2. **h** Transplanted hotspot residues in LD6 in sticks (pink) aligned with p53 peptide residues (teal) in a RMSD of 0.24 Å.

Recently, we described the design of CDH-1 composed of the Bcl-XL:LD3 complex which dissociates in the presence of the Bcl-XL -specific inhibitor A-1155463 (Drug-1). However, Drug-1 is not a clinically-approved drug, which limits the potential translational applications of the CDH-1. We found that LD3 also binds to Bcl2 (Fig. 2a), a protein from the Bcl2 family^25^ closely related to Bcl-XL, with a dissociation constant (K_d_) of 0.8 nM as determined by surface plasmon resonance (SPR) (Fig. 2b). Bcl2 is the target of Venetoclax^26^ (Drug-2) (Supplementary Fig. 1a, b), a BH3 chemical mimetic that blocks the anti-apoptotic Bcl2 protein and is clinically-approved for chronic lymphocytic leukemia (CLL) or small lymphocytic lymphoma (SLL) treatment^21^. We therefore assembled CDH-2, where we exchanged the Bcl-XL component with Bcl2 (Fig. 2a). Drug-2 effectively disrupts the CDH-2 heterodimer in vitro both in an SPR drug competition assay (Fig. 2c, Supplementary Fig. 1c) (IC_50_ = 67.5 nM), as well as by the elution profiles of size-exclusion chromatography coupled with multi-angle light scattering (SEC-MALS) (Supplementary Fig. 1d).

To further expand the panel of CDHs and the chemical space of small-molecule disruptors, we designed a novel CDH-3 (Fig. 2d), based on the starting point of the interaction between p53 and mdm2. Over the past two decades, numerous compounds have been developed to inhibit this interaction and several candidates have been tested in clinical trials^27,28^. Particularly, the dihydroisoquinoline derivative, NVP-CGM097 (Drug-3) (Supplementary Fig. 2a, b), is currently undergoing phase 1 clinical trials^16,22,29^. Leveraging the same computational design approach described for the CDH-1^18^, we utilized the structural information of the mdm2:p53 stapled peptide complex (PDB ID: 5afg)^30^ and searched for proteins that could accommodate the p53 helical motif. We performed a structural search over approximately 11,000 putative protein scaffolds and after the design stage we selected for experimental characterization three designs which we refer to as LD4, LD5 and LD6 (Supplementary Fig. 2c–f, Fig. 1e). From these three designs, LD6 a design based on a thioredoxin protein from mouse^31^, showed the best biochemical behavior being monomeric in solution (Supplementary Fig. 3a) and a melting temperature of 62 ˚C (Supplementary Fig. 3b). LD6 bound to mdm2 with a *K*_d_ of 4 nM (Fig. 2e) as determined by SPR, showing a higher binding affinity than those reported for the wild-type p53_17-29_ peptide (820±60 nM) or the stapled p53 peptide which we used as the input peptide motif (12±3 nM)^30^. Next, we tested if Drug-3 promoted the dissociation of the mdm2:LD6 complex by SPR and determined an IC_50_ of 106 nM (Fig. 2f, Supplementary Fig. 3c). These results were also confirmed using SEC-MALS where the drug dissociated the complex into two monomers (Supplementary Fig. 3d).

To evaluate the structural accuracy of the designed CDH-3, we solved the crystal structure of the mdm2:LD6 complex at 2.9 Å resolution by X-ray crystallography. The structure of the complex closely matched our computational model, with backbone RMSDs of 0.37 Å over the overall structure (Fig. 2g) and 0.24 Å over the p53 motif region (Fig. 2g, Supplementary Fig. 4). The conformation of key residues observed in the native interface were closely mimicked in the crystal structure of LD6 in complex with mdm2. Overall, our results show that we can robustly use computational design to create protein modules which the assembly state is controlled by the presence of small-molecules.

### CDHs function as intra and extra-cellular OFF-switches

**Fig. 3:**
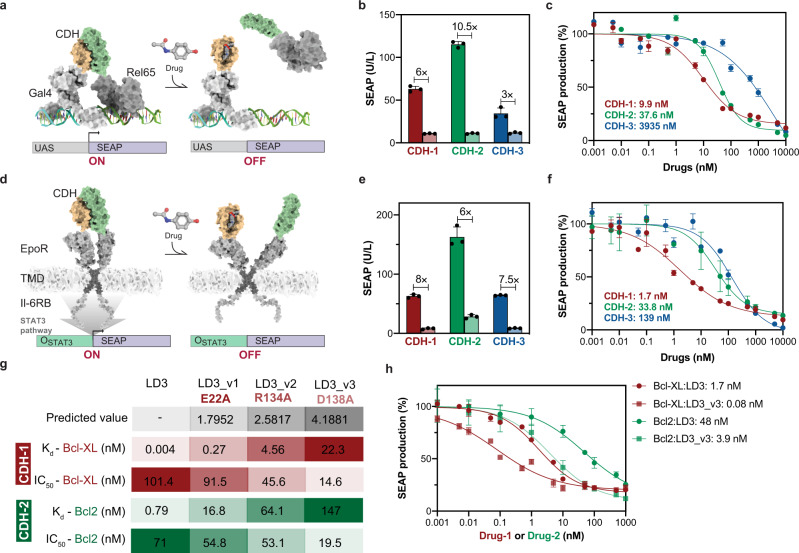
Exploration of distinct cellular activities, localization and affinities of the CDH switches. **a** Schematic representation of the CDHs utilized to control transcription regulation with the GAL4/UAS system (CDH-TFs). **b** CDH-TFs fold-change activity between drug treated (1 µM) versus untreated (DMSO), showing the quantification of SEAP expression after 24 h drug treatment. **c** Drug dose-dependent responses of CDH-TFs quantified by SEAP expression after 24 h drug treatment. **d** Schematic representation of the CDHs utilized to regulate surface signaling receptors (CDH-GEMS) in engineered cells. **e** CDH-GEMS fold-change activity between drug (1 µM) versus no drug treatment (DMSO), showing the quantification of SEAP expression after 24 h. **f** Drug dose-dependent responses of CDH-TFs quantified by SEAP expression after 24 h. **g** Summary of the LD3 mutants including computationally predicted decreases in affinity and experimental measurements using SPR. Values shown are the predicted interaction energy of LD3 mutants (ΔΔG in Rosetta energy units), the binding affinities with Bcl-XL and Bcl2 and IC_50_s of CDH dissociation also for Bcl-XL and Bcl2 with Drug-1 and Drug-2, respectively. **h** Drug dose-dependent responses in engineered cells determined for the CDH-(1-2)-GEMS with LD3 and LD3_v3. The drug receptor utilized were Bcl-XL (CDH-1) in red and Bcl2 (CDH-2) in green, CDHs with original LD3 were in circle symbol and LD3_v3 in square symbol with Bcl-XL and Bcl2. **b**, **c**, **e**, **f**, **h** Each data point represents the mean ± s.d. of three replicates and IC_50_s were computed using four-parameter nonlinear regression. **c**, **f**, **h** Each data point was normalized to the maximal response calculated using four-parameter nonlinear regression. **b**, **c**, **e**, **f**, **h** Source data are available in the source data file.

To test whether the designed CDHs could be useful for different synthetic biology applications, we generated OFF-switches for two cellular systems: transcriptional gene regulation and control of endogenous signaling pathways (Fig. 3a, d). To establish reliable drug concentration ranges for our experiments, we first assessed drug toxicities and impact on the expression of reporter proteins in HEK293T cells, Drugs1-3 were well tolerated up to 10 µM with no detectable changes in the expression of reporter proteins (Supplementary Fig. 5a, b).

To regulate transgene activation events by small molecule inputs we used the CDHs with a split transcription factor design (CDH-TFs) based on the well-characterized Gal4/UAS system^23^ (Fig. 3a).

In brief, the CDH mediated dimerization of the Gal4 DNA binding domain and the Rel65 activation domain triggers the transcription of the reporter protein (secreted alkaline phosphatase (SEAP)). If the CDH switch is functional, the drugs should dissociate the engineered CDH-TFs and diminish SEAP expression. Upon drug treatment, all CDH-(1-3)-TFs were responsive to their respective drugs with dynamic response ranges between 3 and 10-fold (untreated vs 1 μM Drug) (Fig. 3b). We further investigated other parameters that could enhance the dynamic ranges of the CDHs and we observed that increased drug exposure yielded to 8 and 14-fold variations (Supplementary Fig. 6a–c). Additionally, we tested different promoter strengths controlling the CDH-TF’s expression, and observed fold changes of 21 and 13-fold for CDH-1 and CDH-3, respectively (Supplementary Fig. 6d–f). The CDH-TFs showed dose-dependent responses with IC_50_s of 9.6, 33.5 and 540 nM for CDH-1-TF, CDH-2-TF, and CDH-3-TF, respectively (Fig. 3c). The CDH-TF responses were drug-specific, as they only responded to their respective drug (Supplementary Fig. 7a, b). Finally, we explored the dynamic behavior of the designed components by subjecting the CDH-TF transfected cells to intermittent drug treatments. The CDH-TFs showed a significant decrease upon drug treatment and subsequent recovery of SEAP expression upon drug withdrawal (Supplementary Fig. 7c, d), exhibiting a reversible behavior which is central for many synthetic biology applications. Together, these results show that our extended panel of CDHs can be used as intracellular switches and may be adaptable to control several cellular activities in engineered cells that are dimerization/co-localization dependent.

To test modularity and performance robustness of the CDHs in different molecular contexts, we inserted these modules in the generalizable extracellular molecule sensor (GEMS) receptor signaling pathway platform^24^ and built CDH-GEMS. In these constructs the CDHs are localized extracellularly and function as ectodomain switches, fused to the backbone of the erythropoietin receptor, and an intracellular interleukin 6 receptor domain which activates the JAK/STAT pathway (Fig. 3d). The CDH-GEMS trigger the activation of the JAK/STAT pathway by the dimerization of their subunits. Thus, the drugs are expected to split the CDHs inactivating the signaling pathway as assessed by the expression of the reporter protein SEAP. All three CDH-GEMS were responsive to their respective drugs dynamic response range varied from approximately 6-fold (CDH-2) to 8-fold (CDH-1,3) (Fig. 3e). They also showed a dose-dependent response with IC_50_s of 1.7, 23 and 149 nM respectively for CDH(1-3)-GEMS with their cognate drugs (Fig. 3f). The CDH-GEMS only function when both chains are transfected (Supplementary Fig. 7e, f) and are specifically inhibited by their corresponding drugs (Supplementary Fig. 7g). The CDH-GEMS were also reversible upon intermittent drug treatment (Supplementary Fig. 7h, i). Despite that comparisons between CDH-TFs and CDH-GEMS are not straightforward due to differences in the reporter signaling pathways, we observe a general trend for the CDHs to present lower IC_50_s when used as extracellular modules, which is consistent with overcoming drug permeability issues in cells.

Our design process optimized the CDHs for the strength of their interaction, achieving affinities as tight as 4 pM (CDH-1). However, it is unclear how strong the interaction for a specific application should be, and, indeed, many intracellular pathways effectively function with weak protein interaction affinities^32^. We hypothesized that the sensitivity of a CDH could be tuned towards lower drug demand, by weakening the interaction of the two CDH components. We performed computational alanine scanning on the designed binder LD3 to generate lower-affinity binders and selected three mutants (LD3_v1-v3) (Supplementary Fig. 8a–c) which were predicted to decrease the interaction energy (ΔΔG) to different extents (Fig. 3g). LD3 affinity to Bcl-XL measured by SPR was approximately 4 pM and the weakest design (LD3_v3) showed a binding affinity of 22.3 nM, a decrease of more than 5000-fold (Fig. 3g, Supplementary Fig. 8d–f); in vitro IC_50_s also lowered from 101 nM to 14.6 nM, a 7-fold reduction. Next, we tested whether this decrease in affinity also translated into an increased drug sensitivity in CDH-GEMS engineered cells. We observed a 21-fold reduction in IC_50_s of the weakest binder LD3_v3 in Bcl-XL-based CDH-GEMS when compared to the original LD3, showing that we successfully decreased the amount of drug necessary to control the CDH in cells (Supplementary Fig. 8j, Fig. 3h). We also tested the panel of LD3 mutants in the context of the CDH-2 and observed similar trends (Supplementary Fig. 8g–i, k; Fig. 3g, h), using a clinically approved drug. While the overall trends were maintained, the magnitude of the reductions in binding affinities of the CDHs did not directly translate to reductions in cellular activity assays (Supplementary Fig. 9a, b; Fig. 3g), which may reflect a number of factors associated to the complexity of performing measurements in living cells. Full orthogonality of clinical drugs towards cellular targets is challenging to achieve, however these molecular switches function under reduced drug dosages and could avoid potential toxicities while remaining effective at controlling engineered cell activities. In summary, the reduced binding affinity increased the sensitivity of the CDHs towards their small-molecule drugs. This property enlarges the panel of molecular switches that can function under reduced drug dosages and could avoid potential toxicities while remain effective at controlling engineered cell activities.

### Computational design of drug-controlled dimerizing ON switches

CDH components are inherently well suited to trigger OFF outputs, which are highly desirable in some settings, as we previously demonstrated by its integration in CAR-T cells^18^. However, switches that can induce protein colocalization are naturally better suited to obtain ON outputs. We propose that monomerization inducing components (e.g. CDHs) can be rapidly repurposed into dimerization inducing systems (e.g. CIDs), thus diversifying the protein switches available and expanding the chemical space of CID systems to control ON outputs.

**Fig. 4:**
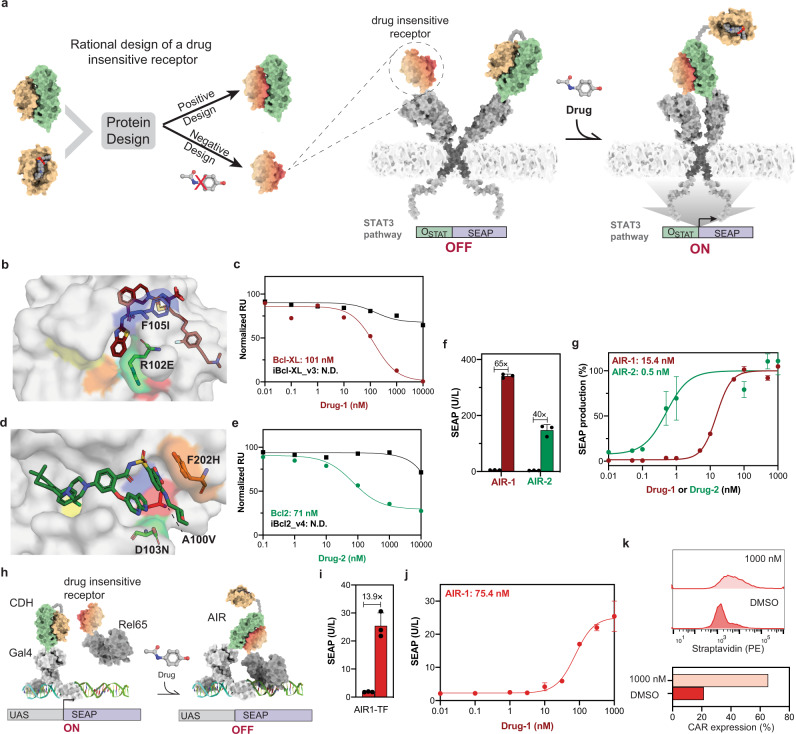
Computational design of protein components to create chemically controlled ON-switches. **a** Computational design approach and architecture of the AIR-GEMS system. Starting from the CDH components (drug receptor (beige) and protein binder (green) we used a multistate design approach to search for drug-receptor variants that retained binding to the protein binder (positive design) and become resistant to the drug (negative design). We then assembled the AIR-GEMS, were the drug triggers the expression of SEAP by activating the JAK-STAT pathway. **b** Structural representation of Drug-1 binding pocket in Bcl-XL. Drug binding pocket (white surface) where the mutations R102E (green sticks) and F105I (blue sticks) were performed to obtain the variant iBcl-XL_v3. Drug-1 is shown in sticks representation and colored in brown. Four other designable residues are highlighted on the surface, E98 in red, T109 in cyan, S145 in orange and A149 in yellow. **c** Apparent IC_50_s for Drug-1 induce dissociation of Bcl-XL:LD3 and iBcl-XL_v3:LD3 determined by SPR drug competition assay. **d** Structural representation of Drug-1 binding pocket in Bcl2. Drug binding pocket (white surface) where the mutations A100V (red sticks), D103N (green sticks) and Y201H (orange sticks) were performed to obtain the variant iBcl2_v4. Drug-2 is shown in sticks representation and colored in green. Two other designable resides are highlighted on the surface, V148 in blue, V156 in yellow. **e** Apparent IC_50_s of Drug-2 induce dissociation of Bcl2:LD3 and iBcl2_v4:LD3 determined by SPR drug competition assay. **f** AIR-GEMS fold-change activity between drug (1 µM) versus no drug treatment (DMSO), showing the quantification of SEAP expression after 24 h. Each bar represents the mean of three biological replicates ± s.d, overlaid with a scatter dot plot of the original data points. **g** Drug dose-dependent responses in engineered cells expressing the AIR-GEMS. Each data point represents the mean ± s.d. of three replicates and the EC_50_s were calculated using four-parameter nonlinear regression. **h** Schematic representation of the AIRs utilized to control transcription regulation with the GAL4/UAS system (AIR-TFs). **i** AIR-1-TF fold-change activity between drug treated (1 µM) versus untreated (DMSO), showing the quantification of SEAP expression after 24 h drug treatment. Each bar shows the mean of three biological replicates ± s.d, overlaid the original data points. **j** Drug dose-dependent responses of AIR-1-TF quantified by SEAP expression after 24 h drug treatment. Each data point represents the mean of *n* = 3 biological replicates, and the EC_50_s were calculated using four-parameter nonlinear regression. **k** AIR-1-TF induces CAR expression comparison between treated (1 µM) versus untreated (DMSO). Histogram of flow cytometry (up) and quantification of CAR expressing cells (bottom). **f**, **g**, **i**, **j**, **k** Source data are available in the source data file.

To address this challenge, we developed a new CID switch architecture, dubbed *activation by inhibitor release* (AIR) switches (Fig. 4a). The AIR architecture relies on three protein components: CDH drug receptor, CDH protein binder and a rationally designed drug-insensitive CDH receptor that retains the binding capability to the protein binder. These three components are assembled into two distinct polypeptide chains to form the AIR switches. In one chain the two elements of the CDH are fused with a flexible peptide linker forming an intramolecular binding interaction that will be disrupted in the presence of the drug, unveiling the binding site of the protein binder (Fig. 4a). In the AIR’s second chain, a drug-insensitive receptor presents the binding site to mediate the intermolecular interaction between the two chains when the drug is present. By creating this multidomain architecture, with tailored components for drug sensitivity, we aim to design protein switches that heterodimerize through an allosteric drug-binding event, effectively mediating a chemically-induced proximity mechanism. Crucial to our AIR architecture is the ability to rationally design drug insensitive receptors that can retain binding activity to the designed binders. In computational design this relates to the well-defined problem of multistate optimization where the sequence space is searched to optimize simultaneously several objective functions^33,34^. In our case, residues in the drug receptor close to the drug and away from the designed binder were selected and sampled for mutations that knocked-out drug binding (*negative* design) and maintained affinity to the protein binder (*positive* design)^35–37^ (Fig. 4a) (see Methods). We first applied this protocol to the CDH-1 (Bcl-XL:LD3), six residues in Bcl-XL (D98, R102, F105, T109, S145 and A149) were selected for multistate design (Fig. 4b), and five putative drug-insensitive Bcl-XL mutants (iBcl-XL_v1-5) were tested (Supplementary Table 2). iBcl-XL_v3 (R102E, F105I) and iBcl-XL_v5 (E98S, F105I) showed the greatest drug resistance (Supplementary Fig. 10a) and did not dissociate from LD3 in the presence of 10 µM of Drug-1, which was sufficient to fully dissociate the wildtype Bcl-XL (Fig. 4c, Supplementary Fig. 10b). The iBcl-XL_v3:LD3 retained a high affinity interaction (*K*_d_ = 3.8 nM) (Supplementary Fig. 10c), which was however considerably lower than Bcl-XL:LD3 complex (*K*_d_ = 4 pM). Structural analysis of the negative design Bcl-XL:Drug-1 complex (PDB id: 4QVX)^19^ shows that F105I, shared by both designs, removes a Pi-stacking interaction with Drug-1, suggesting this mutation as the main resistance driver. To confirm the drug resistance of iBcl-XL_v3 in a cellular context, we tested the iBcl-XL_v3 paired with LD3 in the CDH-GEMS platform. In contrast to the CDH-1-GEMS, Drug-1 failed to disrupt the iBcl-XL_v3:LD3 complex at µM concentrations (Supplementary Fig. 10d).

Next, we assembled the AIR sensors in the GEMS platform (Fig. 4a, right) to evaluate its potential as an effective ON switch. We fused the AIR components in the ectodomains of the GEMS and measured SEAP activity as a reporter for drug-triggered activation. We constructed the AIR-1-GEMS using iBcl-XL_v3 in one chain and a fused CDH-1 (LD3-GGGGSX3 linker-Bcl-XL) in the second chain. We co-expressed these constructs in HEK293T cells, and observed that the AIR-1-GEMS was responsive to Drug-1, effectively turning ON the expression of the reporter gene SEAP. Furthermore, the AIR switches showed a very sensitive drug response (EC_50_ = 18.68 ± 4.65 nM) (Fig. 4g), and a 64-fold dynamic response range (untreated vs 100 nM Drug-1 treated) (Fig. 4f).

To test whether we could design a second ON-switch controlled by a different drug, we applied the same design strategy to generate a Drug-2 resistant receptor and created the AIR-2 based on the CDH-2 components (Bcl2:LD3) which is responsive to the clinically approved drug Venetoclax (Drug-2). Similarly to the AIR-1, five residues located in the drug binding site (A100, D103, V148, V156, Y202) (Fig. 4d) were used for multistate design to create drug-insensitive Bcl2 variants (iBcl2). Four designs (Supplementary Table 3) were screened on the AIR-GEMS platform and one showed activation upon addition of Drug-2 (Supplementary Fig. 11a). We refer to this switch as the AIR-2-GEMS, which was composed of iBcl2_v4 (A100V, D103N, Y202H) and the fused CDH-2 (LD3-GGGGSX3 linker-Bcl2), and showed an EC_50_ of 0.5 ± 0.27 nM (Fig. 4g). Subsequently we verified at the biochemical level that it retained the interaction with LD3 (K_d_ = 9.8 µM) (Supplementary Fig. 11b) and was resistant to Drug-2 both in vitro (Fig. 4e, Supplementary Fig. 11c) as well as in cell-based assays (Supplementary Fig. 11d). A comparison between the two AIR-GEMS shows that AIR-2-GEMS is approximately 36-fold more sensitive to drug treatment in terms of EC_50_s, but AIR-1-GEMS shows a higher magnitude of response when comparing the resting and the fully activated states (350 U/L SEAP in AIR1-GEMS vs 160 U/L in AIR2-GEMS at 100 nM Drug) (Fig. 4f). These distinct behaviors may be due to differences in affinities of LD3 to the different drug receptors, where a lower affinity affords a more responsive receptor requiring lower drug concentrations, but the output is less sustained reaching overall lower activation levels.

To further expand the usage of the AIRs we engineered split transcription factor systems (AIR-TFs) similar to the CDH-TFs (Fig. 4h). Gal4 DNA binding domain with genetically fused CDH-1 and drug insensitive receptor iBclxL_v3 with the Rel65 activation domain were assembled. AIR-1-TF was activated by Drug-1 showing a dynamic response range of 13 fold change (Fig. 4i) and a dose-dependent response with EC_50_ of 75 nM (Fig. 4j). Furthermore, we applied the AIR-1-TF to control the expression of a therapeutically relevant transgene expression. One approach to modulate the activity of CAR-T cells is to control the expression of the CAR^38^, we sought use the AIR-1-TF to obtain the chemically-induced CAR expression. We observed that the AIR-1-TF could effectively induce the CAR expression in the presence of Drug-1, showing a robust increase in the CAR-expressing cell population (Fig. 4k, Supplementary Fig. 12).

These results suggest that the conversion of CDH components into the AIR architecture is an effective way to design ON switches responsive to low-doses of small-molecule drugs (including clinically approved). As the panel of clinically-tested PPI inhibitors grows, our AIR design strategy could be a general approach to expand the panel protein switches to control cellular activities.

### Multi-input multi-output control in engineered cells using orthogonal switches

An overarching goal in synthetic biology is to use living cells as bio-computing units, where orthogonal sensing components display distinct switching behaviors^39^. The need for these complex logic devices goes beyond basic applications, as shown in the CAR-T cell field, where there is a growing need to control multiple functions using orthogonal signals (e.g., suicide switches^40^, tunable activity^41^, molecule secretion^42^). As a final proof-of-concept, we engineered multi-input/multi-output cells by combining several designed switches in HEK293T cells, as a model system for further applications. In analogy with control systems in electrical engineering, we sought to implement three distinct input/output systems beyond single-input/single-output (SISO), such as: multi-inputs/single-output (MISO), single-input/multi-outputs (SIMO) and multi-inputs/multi-outputs (MIMO)^43^.

**Fig. 5:**
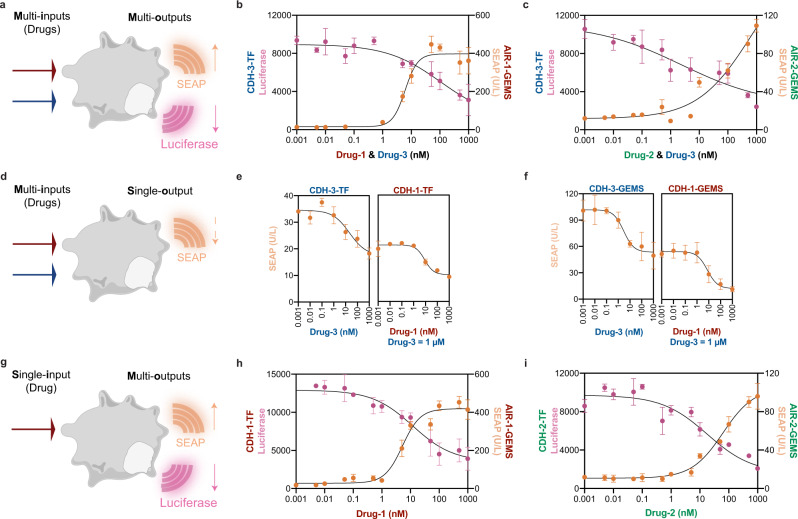
Orthogonal chemical switches enable implementation of multi-input multi-output control modes in mammalian cells. **a** Scheme of multi-inputs multi-outputs (MIMO) designer cells where two drugs control two different outputs. **b**, **c** Cells were co-transfected with AIR-1/2-GEMS and CDH-3-TF regulate SEAP and Luciferase expression, respectively. Drug concentrations ranged from 0.001 nM to 1 µM and different combinations were added depending on the protein components, Drug-1 + Drug-3 (**b**) and Drug-2 + Drug-3 (**c**). **d** Scheme of multi-inputs single-output (MISO) designer cells where two drugs control one output. **e**, **f** Quantification of SEAP activity controlled by CDH-1-TF and CDH-3-TF circuits (**e**) or CDH-1-GEMS and CDH-3-GEMS (**f**). Drug-3 concentrations ranged from 0.001 nM to 1 µM and Drug-1 treatments were performed in the presence of 1 µM Drug-3. **g** Scheme of single-input multi-outputs (SIMO) designer cells where one drug controls two outputs. **h**, **i** Quantification of SEAP and Luciferase activities under Drug-1 (**h**) or Drug-2 (**i**). AIR-1/2-GEMS coupled with SEAP expression circuits were co-transfected with CDH-1/2-TF circuits which control the Luciferase production. Respective drug concentrations ranged from 0.001 nM to 1 µM. All values presented are mean ± s.d. of three replicates and curves were fitted by four-parameters nonlinear regression. **b**, **c**, **e**, **f**, **h**, **i** Source data are available in the source data file.

We first engineered MIMO cells to respond to two drugs with distinct outputs (Fig. 5a) by co-transfecting the CDH-3-TF with either AIR-1-GEMS (Fig. 5b) or AIR-2-GEMS (Fig. 5c). The combination of two drugs did not affect cell viability at the highest concentration of 1 µM each (Supplementary Fig. 13a). The CDH-3-TF luciferase reporter expression decreased in a Drug-3 dose-dependent manner and AIR-GEMS (AIR-1 and AIR-2) showed an increase of SEAP expression dependent on their cognate drugs (Fig. 5b–c). These MIMO circuits showed an orthogonal control of two distinct cellular activities without any detectable non-specific regulation by the two drug inputs (Supplementary Fig. 13b–e).

We then engineered MISO cells (Fig. 5d) with CDH-1-TF and CDH-3-TF dual switches (Fig. 5e) that encode OFF behaviors controlled by Drug-1 and Drug-3. As expected, the individual CDHs showed similar response curves to those observed when they were tested in isolation (Fig. 2c). Interestingly, when treated with both drugs the engineered cells showed a unique behavior where the MISO’s response curve showed three persistent response levels (high, medium, low) controlled by drug amounts (Fig. 5e, Supplementary Fig. 13f). This output behavior is distinct from the typical SISO systems which only show two persistent states (high, low) (Fig. 2c, f). Similarly, the engineered cells endowed with CDH-1-GEMS and CDH-3-GEMS dual-circuits (Fig. 5f, Supplementary Fig. 13g) showed three persistent response levels. This type of output in biological systems could be utilized to produce graded levels of responses that are robust to minor oscillations in drug concentrations, and could be used to dissect fundamental biological processes but also to provide persistent response levels in translational applications.

Finally, we engineered SIMO cells (Fig. 5g) using two designed switches that produce ON and OFF responses upon exposure to the same drug. We transfected cells with combinations of AIR-GEMS and CDH-TFs which controlled the expression of the reporter proteins SEAP and Luciferase, respectively. Both Drug-1 and Drug-2 simultaneously activated SEAP production and suppressed the expression of Luciferase (Fig. 5h, i) in their respective switch systems, demonstrating that we successfully designed protein components which can physically dissociate and associate under the control of the same drug. To the best of our knowledge, Drug-1 and Drug-2 are the only two small molecules that like rapamycin can control both assembly^4,44^ and disassembly^15^ of protein components. Therefore, computational design expanded the repertoire of protein switches controlled by the same compounds that can be used to simultaneous turn ON and OFF synergistic outputs.

In summary, leveraging our computationally designed switches we created cellular bio-computing units with multiple output modalities that can be useful to deal with the inherent complexity of engineering mammalian cells and have the potential for translational applications in synthetic biology.

## Discussion

Protein components to chemically control cellular activities are the basis of many applications in synthetic biology. However, the panel of protein switches available to control the assembly of protein complexes for synthetic biology remains extremely limited^1^. Here, we presented a structure-based design blueprint to repurpose PPIs with known inhibitors into protein switches that can mediate both assembly and disassembly of hetero-dimeric complexes.

Specifically, we presented three CDH switches, controlled by three different drugs, along with multiple tailor-made affinity variants. Some of our observations on tuning CDH affinities highlight the challenge of designing components for acting in complex biological systems. For instance, by weakening the CDH interface, we can tune the affinity of the dimer down to 5000-fold weaker. Yet despite this substantial difference in binding affinity, we find that the changes in cell-based measurements are much smaller (11 to 26-fold). Thus, we find a non-linear relationship between hetero-dimer affinities and drug IC_50_s in cells, pointing to the need to test a range of switches to optimize drug responsiveness. The CDHs can be utilized in the context of diverse proximity-based signal components intra- and extra-cellularly, which shows their modularity and wide applicability. Furthermore, CDH-2 (Bcl2:LD3) is controlled by an FDA-approved drug, Venetoclax and was optimized toward lower drug demand, which could reduce risks of drug-induced toxicity or off-target effects.

In an effort to create chemically mediated dimerization switches, we further devised a multidomain architecture (referred to as AIR switches), for which we computationally designed a drug insensitive receptor that retained the ability to bind with the designed protein binder. The AIR switches showed an exquisite response to the drug inputs and we foresee that this design strategy will be broadly applicable to design other chemically controlled heterodimers, enriching this important class of protein components in synthetic biology. This expanded repertoire of CDHs and AIRs provides the opportunity to engineer more elaborated cellular outputs. As a proof-of-concept, our designed switches were combined in engineered cells which displayed several multi-input/multi-output behaviors. One interesting control mode was achieved where a single input drug regulated multiple outputs by exploiting the same small-molecule to trigger ON- and OFF- responses simultaneously. This type of protein circuitry where the same small molecule can toggle between two antagonistic cellular outputs, may be a promising option for instance to control safety and efficacy in engineered cells.

Altogether, CDHs and AIRs enable a generalizable approach to develop controllable modules for synthetic biology applications. We envision that the computational protein design toolbox will help to bridge the gap between the engineering of protein components and next-generation cell-based therapies^45^.

## Methods

### Computational design of CDH-3

The design of CDH-3 followed a similar protocol to that presented in our previous work^18^, based on a side chain grafting approach. The mdm2:p53 peptide interaction was selected as a starting point, since multiple small molecule inhibitors bind to the mdm2 receptor protein and prevent binding of p53. An 8 amino acid helical fragment (**F**XXX**W**XX**L**, where F, W, and L are hotspot residues and X are the designable residues) was extracted from the helical binding motif of an mdm2-binding, p53-mimic, stapled peptide (PDB id: 5afg) and selected as the ‘binding seed’. A database of monomeric protein structures obtained through X-ray crystallography was assembled, where proteins were sourced from the Protein Databank (PDB) if they met the following: (a) proteins with a global stoichiometry assigned in the PDB as monomers, (b) an amino acid sequence length between 80 and 160, (c) proteins containing helical secondary structures. The computational design protocol was executed as a script using RosettaScripts, and entailed the following steps. The MotifGraft^46^ program in Rosetta attempted to graft the binding seed to all proteins in the database (*scaffold* proteins). Proteins on which the fragment was grafted to a similar backbone fragment, with a maximum C_α_ root mean square deviation of 1.0 Å, and where they maintained a steric compatibility (a clash score of maximum 5 Rosetta Energy Units or REU) with mdm2, were accepted. Once scaffolds were matched, residue positions surrounding the binding seed were designed using the Rosetta fixed backbone design^47,48^, allowing amino acid mutations to residues with a positive score in the BLOSUM62 matrix^49^, with respect to the amino acid identity in the wildtype protein. The restriction to mutations based on the BLOSUM62 matrix was performed to prevent mutations to the original scaffold that could affect its stability, folding pathway, or solubility. The resulting designed proteins were scored using Rosetta for the complex energy (ΔΔG) predicted score, buried solvent accessible area and unsatisfied hydrogen bonds. Designs where Rosetta’s ΔΔG energy was higher than -7 REU were discarded. A visual inspection of the resulting scaffolds showed that many of them had non-globular structures, with extended conformations and poor packing of the binding seed. To remove scaffolds with non-globular structures, we computed a globularity score on proteins based on a metric created by Miller et al^50^, which found that the mass *M* of globular proteins correlates with the solvent accessible surface area *A* under the power law:

$${{{{{{\rm{A}}}}}}}_{{{{{{\rm{s}}}}}}}=6.3{{{{{{\rm{M}}}}}}}^{0.73}$$

We thus computed a globularity score *G* = *A*_s_/6.3*M*^0.73^, and scaffolds with *G* < *0.9* were discarded from consideration. Finally, scaffolds were visually inspected, and those where a large small molecule ligand binding site was present in the original structure near the novel interface, or those where the binding seed was grafted to a terminal region of the protein, were excluded. We thus selected 3 protein scaffolds, LD4, (designed from scaffold hydroquinone flavodoxin from Desulfovibrio vulgaris with PDB id: 1AKU), LD5 (designed from scaffold with PDB id: 2IFQ) and LD6 (designed from scaffold with PDB id: 2FWE). After close inspection of LD6, residue 23 was manually mutated to Tyr, as this was the identity found in the starting stapled peptide.

### Protein expression and purification

The genes encoding all the designs were purchased from Twist Bioscience. Genes were synthesized with N-terminal 6×His-tag and cloned into the pET11b vector using Gibson assembly (New England Biolabs, E2611S). The plasmids were transformed into XL10 gold *E. coli* for plasmid amplification and BL21(DE3) *E. coli* (Thermo Fisher) for protein expression. A single clone was picked to inoculate 500 ml of Terrific Broth (Merck Millipore, 101629) containing ampicillin (100 µg/ml). The cultures were grown at 37 °C until OD_600_ reached around 0.8 and protein expression was induced with 1 mM IPTG at 20 °C. After overnight induction, cells were pelleted by centrifugation at 3800 g. The harvested pellet (thawed on ice, if frozen after centrifugation) was resuspended in 40 ml lysis buffer (50 mM Tris, 500 mM NaCl and 5% Glycerol in pH 7.5) supplemented with 100 µg/ml PMSF (ROTH, 6376.2). The slurry was sonicated for 30 mins, centrifuged at 20,000 g for 20 mins, and the supernatant was collected. For mdm2 protein, the protein was extracted from inclusion bodies after centrifugation. The collected pellet was washed twice with 50 ml lysis buffer containing 0.05% Triton-X100 (AppliChem) and solubilized by 20 ml lysis buffer supplemented with 8 M urea (Merck, U4883) and 10 mM β-mercaptoethanol (AppliChem, A4338.0100). Resuspended inclusion bodies were dialyzed against 1 liter of 4 M guanidine hydrochloride in pH 3.5 supplemented with 10 mM β-mercaptoethanol. Next, the protein was refolded by dropwise addition with 1 liter of 10 mM Tris-HCl (pH 7.0) containing 1 mM EDTA and 10 mM β-mercaptoethanol and slowly mixed overnight at 4 °C. The supernatant and the refolded solution were loaded to AKTA pure system (GE Life Science) for nickel affinity purification and the target protein was eluted by elution buffer (50 mM Tris, 500 mM NaCl, 300 mM imidazole). The eluted protein was further purified by gel filtration to separate the monodisperse population. The purified proteins were concentrated, aliquoted and stored at −80 °C.

### Compounds

Venetoclax (>99.9%, Chemietek CT-A199), A1155463 (99.5%, Chemietek CT-A115) and NVP-CGM097 (100% optically pure, Chemitek CT-CGM097), were directly used without further purification. Venetoclax, A1155463, and NVP-CGM097 were each dissolved in DMSO as 10 mM stocks. Stocks were aliquoted and stored at −20 °C until use.

### Circular dichroism spectrum

Folding and thermostablility of LD6 was measured using circular dichroism spectroscopy at ramping temperatures (25–90 °C). Protein samples were dissolved in phosphate saline buffer at a protein concentration around 0.2 mg/ml (20 µM). The sample was loaded into 0.1 cm path-length quartz cuvette (Hellama). The far-UV CD spectrum between 190 nm and 250 nm was recorded with a Chirascan V100 spectrometer with a band width of 2.0 nm, and scanning speed was at 20 nm/min. Response time was set to 0.125 s and spectra were averaged from 2 individual scans.

### Size-exclusion chromatography coupled with multi-angle light scattering

LD6 was further characterized by Size Exclusion Chromatography coupled to Light Scattering (SEC-MALS) for solution behavior, and to study dimerization and drug-induced monomerization properties. LD6 was injected at 50–100 µM into a Superdex^TM^ 75 300/10 GL column (GE Healthcare) using a high-performance liquid chromatography system (Ultimate 3000, Thermo Scientific) at a flow rate of 0.5 ml/min. The UV spectrum at 280 nm was collected along with static light scatter signal by a MALS device (miniDAWN TREOS, Wyatt). For determining drug-induced monomerization, 50 µM mdm2 or Bcl2 were mixed with equal molarity of LD6 and LD3, respectively. Then the mixtures were treated with either 100 µM (two-fold excess) of the corresponding drugs or the same volume of DMSO to detect complex formation and forced dissociation in SEC-MALS. The light scatter signal of the sample was collected from three different angles and the results were analyzed using the ASTRA software (version 6.1, Wyatt).

### Surface plasmon resonance for assessing protein–protein binding affinity

Surface plasmon resonance (SPR) measurements were performed on a Biacore 8 K device (GE Healthcare). Drug-receptor proteins (Bcl-XL, Bcl2 and mdm2) were immobilized on a CM5 chip (GE Life Science) as a ligand with the concentration at 5 µg/ml for 120 seconds contact time in pH 4.0 (mdm2) or pH 4.5 (Bcl-XL and Bcl2) sodium acetate solutions, respectively. Serial dilutions of the analytes in HBS-EP buffer (10 mM HEPES, 150 mM NaCl, 3 mM EDTA and 0.005% Surfactant P20; GE Life Science) were flown over the immobilized chips. After each injection cycle, surface regeneration was performed using 10 mM NaOH (pH 11.95). Affinity (K_d_) and kinetic parameters (*K*_on_ and *K*_off_) were obtained using a 1:1 Langmuir binding model with Biacore 8 K evaluation software.

### SPR drug competition assay

Drug IC_50_s of disrupting CDH heterodimers were measured on a Biacore 8 K device. 4 µM of LD3 and its variants (LD3_v1-3) or LD6 were mixed with their respective drugs. Drug concentrations ranged from 10 µM to 0.01 nM in 10x serial dilutions. The drug-binder mixtures were injected on the drug-receptor protein (Bcl-XL, Bcl2 or mdm2 as indicated) immobilized channel. Multiple-cycle injection of the protein-drug complex with different stoichiometry were performed to measure the decrease of maximal RUs (response units at 120 sec). Apparent IC_50_s were obtained using the inhibition versus response fitting models in prism software (Version 8.3.0).

### Purification of mdm2 and LD6 for X-ray crystallography

The complex of mdm2 with LD6 was prepared by mixing each of the components at equal molar ratio. After overnight incubation, the complex was purified by size exclusion chromatography using a Superdex75 16 600 (GE Healthcare) equilibrated in 10 mM Tris pH 8, 100 mM NaCl and subsequently concentrated to ~15 mg/ml (Amicon Ultra-15, MWCO 3,000). Crystals were grown using the sitting-drop vapor-diffusion method at 291 K in a solution containing 1.5 M Ammonium sulfate, 0.1 M Sodium cacodylate at pH 6.5. For cryo protection, crystals were briefly swished through mother liquor containing 25% glycerol. Diffraction data were recorded with a X06DA (PXIII) beamline at the Paul Scherer Institute, Switzerland. The diffracted crystal of mdm2/LD6 belonged to space group P4_3_22. Data was integrated and processed to 2.9 Å with a high-resolution cut at I/σ = 1 applied by the X-ray Detector Software (XDS)^51^. The structure was determined by molecular replacement using the Phenix Phaser module^52^. The searching of the initial phase was performed by using the mdm2 structure (PDB id: 5AFG) and the computationally designed model LD6 as a search model. Manual model building was performed using Coot^53^, and automated refinement using Phenix Refine^54^. The final refinement statistics are summarized in Supplementary Table 1.

### CCK8 cell viability assay

A cell counting kit-8 (CCK-8) assay (Sigma) was used to measure the cytotoxicity of three drugs on HEK293T cells. 10,000 cells per well were pre-seeded into 96 well plates in 100 µl complete culture medium. The culture medium was changed into drug containing medium with drug concentrations of 0 µM (0.1% DMSO),1 µM, 5 µM and 10 µM. After 24 h drug incubation, 10 µl WST-8 solution was added to each well for 4 h incubation under standard conditions. The absorbance at 450 nm was determined by a multiplate reader (Tecan Infinite 500).

### Cell transfection and drug treatment

HEK293T cells were maintained in DMEM medium with 10% FBS (Gibco) and Pen/Strep (Thermo Fisher). Cells were maintained and split every two days at around 80% confluence. HEK293T cells were seeded 24 h before transfection, and transfected with the lipofectamine 3000 kit (Thermo Fisher). Plasmids were cotransfected according to the Table S7. For the drug OFF-switch experiments, drugs were added 12 h post transfection and incubated with cells for 24 h before the SEAP detection assay.

### SEAP detection assay

Secreted alkaline phosphatase (SEAP) activity (U/L) in cell culture supernatants was quantified by kinetic measurements at 405 nm (1 min/measurement for 30 cycles) of absorbance increase due to phosphatase-mediated hydrolysis of para-nitrophenyl phosphate (pNPP). 4–80 µl of supernatant was adjusted with water to a final volume of 80 µl, heat-inactivated (30 min at 65 °C), and mixed in a 96-well plate with 100 μL of 2 × SEAP buffer (20 mM homoarginine (FluorochemChemie), 1 mM MgCl_2_, 21% (v/v) diethanolamine (Sigma Aldrich, D8885), pH 9.8) and 20 μl of substrate solution containing 20 mM pNPP (Sigma Aldrich, 71768).

### Reversibility assay (Dynamic regulation)

Cells were pre-seeded 24 h before the transfection in 12-well plate. The ON-OFF-ON mode was grew without drug in the first 36 h and passed 1/3 of cells to the new dish with 500 nM drugs supplemented for next 36 h with refreshing the drugs every 12 h, then passed to the new dish with the removal of drugs. The OFF-ON-OFF mode was treated with 500 nM drugs 12 h post-transfection for the following 36 h. Cells were also passed every 36 h and cultured in the absence of drug for the hours from 36 to 72 h, then add drugs again from 72 h. SEAP samples were taken every 12 h from the culture supernatant.

### Design of weaker affinity variants

The LD3 protein was computationally redesigned for decreased binding to Bcl-XL with a range of decreasing affinities. Rosetta’s alanine scanning filter was used to evaluate the change in ΔΔG for the LD3:Bcl-XL complex (PDB id: 6IWB) upon mutating each of the 22 residues in the interface of LD3 to Alanine. The resulting list was then sorted by the change in ΔΔG, and three residues with positive levels of change in ΔΔG were selected: E22 (1.8 REU), R134 (2.6 REU) and D138 (4.2 REU), where higher REU values are predicted to result in greater affinity losses. The three mutations were selected to provide a ‘gradient’ of affinities between LD3 and Bcl-XL.

### Drug-insensitive receptor mutations predictions

Bcl-XL and Bcl2 were redesigned for resistance to Drug-1 and Drug-2, respectively, following a computational strategy similar to one used to predict drug resistance^33,34^. Briefly, a set of residues in the receptor protein’s (Bcl-XL/Bcl2) binding site was selected for redesign. From this set, a number of mutations was evaluated for binding energy to the binder protein (LD3) (positive design) or the drug (negative design). Afterwards all mutations were ranked according to the difference in energy between the positive design and negative design. Bcl-XL**:** The structure of Bcl-XL bound to Drug 1 (A-1155463, PDB id: 4QVX) was used for the negative design strategy, while the model of Bcl-XL bound to LD3 (based on the Bcl-XL:BIM BH3 structure with PDB id: 3FDL) was used for positive design. Six Bcl-XL residues in the binding site of Drug-1 (E98, R102, F105, T109, S145 and A149) were manually selected for redesign due to their proximity to drug moieties and relative distance to LD3 in the positive design structure. Each of these residues was allowed to mutate to residues with similar size/properties, restricted to a maximum of two simultaneous mutations from wildtype: E98: {E/S}, R102: {F/R/K/D/E/H}, F105:{F/L/V/I/A}; T109: {S/A/T/L/V}; S145: {S/D/E/V/A}; A149:{V/A/L/I}. The total sequence space thus consisted of 253 unique sequences. The Rosetta program was then used to redesign the positive design structure (LD3:Bcl-XL complex) and the negative design structure (Drug-1:BclxL complex) for each of the 253 sequences. A score was computed for the complex state of each sequence in each of the two states, and sequences were ranked according to the ratio of the positive design (bound) score - negative design (bound) score (Supplementary Table 2). Five sequences iBcl-XL_v1 (T109L, A149L), iBcl-XL_v2 (A149V), iBcl-XL_v3 (R102E, F105I), iBcl-XL_v4 (R102F, T109V), and iBcl-XL_v5 (E98S, F105I) were selected from the top results. The structure of Bcl2 bound to Drug-2 (Venetoclax, PDB id: 6O0K) was used for the negative design strategy, while the model of Bcl2 bound to LD3 (PDB id: 6IWB) was used for positive design. Five Bcl2 residues in the binding site of Drug-2 (A100, D103, V148, V156 and Y202) were manually selected for redesign due to their closeness to drug moieties and relative distance to LD3 in the positive design structure. Each of these residues was allowed to mutate to amino acids with similar size/properties, restricted to a maximum of two simultaneous mutations from wildtype: A100: {A/S/T/V}, D103: {D/N/E/Q/S}, V148: {V/I/L/M/T}, V156: {V/I/L/M/T} and Y202: {Y/W/F/H/R/K/Q/E}. The total sequence space thus consisted of 251 unique sequences. The Rosetta program was then used to redesign the positive structure (PDB id: 6IWB) and the negative design structure (PDB id: 6O0K) for each of the 251 sequences. A score was computed for the complex state of each sequence in each of the two states, and sequences were ranked according to the ratio of positive design score/negative design score (Supplementary Table 3). Three sequences iBcl2_v1(156I, 202H), iBcl2_v2(103 N, 202H) and iBcl2_v3(100T_103S) were selected from the top results. Three additional mutations were enriched in the top designs, and therefore we designed a further version, iBcl2_v4 (100V_103N_202H).

### Flow cytometry of surface expression of anti-HER2 CAR

HEK293T cells were transfected with AIR-1-TF inducible anti-HER2 CAR expression system, and treated with indicated concentration of drugs to induce the anti-HER2 CAR expression. Cells were trypsinized, harvested, and washed twice by FACS buffer (PBS with BSA albumin (0.2%, wt/v, Sigma-Aldrich)) after 24 h drug incubation. Surface staining of 4D5 scFv anti-HER2 CAR using biotinylated human Her2/ErbB2 Protein (Acro Biosystems) with a ratio of 1:100 at 4 °C for 15 mins. and avidin-Alexa Fluor PE conjugate (Invitrogen/Thermo Fisher Scientific) 24 h post drug induction. Cells were centrifuged and washed by FACS buffer to remove the remaining HER2/ErbB2 protein, then staining with avidin-Alexa Fluor PE conjugated (Bioscience) in a ratio of 1:100 at 4 °C for 15 mins. Cells were washed twice and resuspended in the FACS buffer for flow analysis.

### Statistics

Apparent IC_50_s of SPR drug competition assay were calculated using three-parameter nonlinear regression in GraphPad Prism (Version 8.3.0). Representative data of cellular assays are presented as individual values and mean values (bars). *n* = 3 refers to biological replicates. All IC_50_/EC_50_ values of cellular assays (CDH-TF, CDH-GEMS, AIR-TF and AIR-GEMS) reported were calculated using four-parameter nonlinear regression ± s.d.

### Reporting summary

Further information on research design is available in the Nature Research Reporting Summary linked to this article.

## Supplementary information

Supplementary Information

Reporting Summary

## Data Availability

The data supporting the findings of this study are available within the article and its Supplementary Information. Source data is provided with this paper. Coordinates of the determined structure have been deposited in the PDB with accession code 7AYE. Designed protein sequences are provided in supplementary Tables 4–5. Plasmids encoding the CDH and AIR components available from Addgene (Addgene ID: 174549-174553). Other data and reagents are available from the corresponding authors upon reasonable request. Source data are provided with this paper.

## Source data

Source Data

## Code availability

All code used to perform the design simulation can be found at: https://github.com/LPDI-EPFL/CDH_AIR and cited with: 10.5281/zenodo.5155700.

## References

[1] Stanton BZ, Chory EJ, Crabtree GR (2018). Chemically induced proximity in biology and medicine. Science.

[2] Meng F, Ellis T (2020). The second decade of synthetic biology: 2010–2020. Nat. Commun..

[3] Stapornwongkul KS, de Gennes M, Cocconi L, Salbreux G, Vincent J-P (2020). Patterning and growth control in vivo by an engineered GFP gradient. Science.

[4] Wu C-Y, Roybal KT, Puchner EM, Onuffer J, Lim WA (2015). Remote control of therapeutic T cells through a small molecule-gated chimeric receptor. Science.

[5] Barlow AD, Nicholson ML, Herbert TP (2013). Evidence for rapamycin toxicity in pancreatic β-cells and a review of the underlying molecular mechanisms. Diabetes.

[6] Sarkar S, Ravikumar B, Floto RA, Rubinsztein DC (2009). Rapamycin and mTOR-independent autophagy inducers ameliorate toxicity of polyglutamine-expanded huntingtin and related proteinopathies. Cell Death Differ..

[7] Sun J, Sadelain M (2015). The quest for spatio-temporal control of CAR T cells. Cell Res..

[8] Riddell SR (1996). T–cell mediated rejection of gene–modified HIV–specific cytotoxic T lymphocytes in HIV–infected patients. Nat. Med..

[9] Jensen MC (2010). Antitransgene rejection responses contribute to attenuated persistence of adoptively transferred CD20/CD19-specific chimeric antigen receptor redirected T cells in humans. Biol. Blood Marrow Transpl..

[10] Berger C, Flowers ME, Warren EH, Riddell SR (2006). Analysis of transgene-specific immune responses that limit the in vivo persistence of adoptively transferred HSV-TK–modified donor T cells after allogeneic hematopoietic cell transplantation. Blood.

[11] Hill ZB, Martinko AJ, Nguyen DP, Wells JA (2018). Human antibody-based chemically induced dimerizers for cell therapeutic applications. Nat. Chem. Biol..

[12] Foight GW (2019). Multi-input chemical control of protein dimerization for programming graded cellular responses. Nat. Biotechnol..

[13] Glasgow AA (2019). Computational design of a modular protein sense-response system. Science.

[14] Rivera VM (2000). Regulation of protein secretion through controlled aggregation in the endoplasmic reticulum. Science.

[15] Rollins CT (2000). A ligand-reversible dimerization system for controlling protein-protein interactions. Proc. Natl Acad. Sci. USA.

[16] Ran X, Gestwicki JE (2018). Inhibitors of protein–protein interactions (PPIs): an analysis of scaffold choices and buried surface area. Curr. Opin. Chem. Biol..

[17] Arkin MR, Tang Y, Wells JA (2014). Small-molecule inhibitors of protein-protein interactions: progressing toward the reality. Chem. Biol..

[18] Giordano-Attianese G (2020). A computationally designed chimeric antigen receptor provides a small-molecule safety switch for T-cell therapy. Nat. Biotechnol..

[19] Tao Z-F (2014). Discovery of a potent and selective BCL-XL inhibitor with in vivo activity. ACS Med. Chem. Lett..

[20] Deeks ED (2016). Venetoclax: first global approval. Drugs.

[21] Roberts AW (2016). Targeting BCL2 with venetoclax in relapsed chronic lymphocytic. Leuk. N. Engl. J. Med..

[22] Holzer P (2015). Discovery of a dihydroisoquinolinone derivative (NVP-CGM097): a highly potent and selective MDM2 inhibitor undergoing phase 1 clinical trials in p53wt tumors. J. Med. Chem..

[23] Tang JCY (2013). A nanobody-based system using fluorescent proteins as scaffolds for cell-specific gene manipulation. Cell.

[24] Scheller L, Strittmatter T, Fuchs D, Bojar D, Fussenegger M (2018). Generalized extracellular molecule sensor platform for programming cellular behavior. Nat. Chem. Biol..

[25] Hanada M, Aimé-Sempé C, Sato T, Reed JC (1995). Structure-function analysis of Bcl-2 protein. Identification of conserved domains important for homodimerization with Bcl-2 and heterodimerization with Bax. J. Biol. Chem..

[26] Souers AJ (2013). ABT-199, a potent and selective BCL-2 inhibitor, achieves antitumor activity while sparing platelets. Nat. Med..

[27] Neochoritis, C., Estrada-Ortiz, N., Khoury, K. & Dömling, A. p53–MDM2 and MDMX antagonists. In *Annual Reports in Medicinal Chemistry* vol. 49 167–187 (Elsevier, 2014).

[28] Tisato V, Voltan R, Gonelli A, Secchiero P, Zauli G (2017). MDM2/X inhibitors under clinical evaluation: perspectives for the management of hematological malignancies and pediatric cancer. J. Hematol. Oncol. J. Hematol. Oncol..

[29] Townsend EC (2015). The MDM2 inhibitor NVP-CGM097 is highly active in a randomized preclinical trial of B-cell acute lymphoblastic leukemia patient derived xenografts. Blood.

[30] Lau YH (2015). Double strain-promoted macrocyclization for the rapid selection of cell-active stapled peptides. Angew. Chem. Int. Ed..

[31] Stirnimann CU (2006). High-resolution structures of *Escherichia coli* cDsbD in different redox states: a combined crystallographic, biochemical and computational study. J. Mol. Biol..

[32] Hein MY (2015). A human interactome in three quantitative dimensions organized by stoichiometries and abundances. Cell.

[33] Havranek JJ, Harbury PB (2003). Automated design of specificity in molecular recognition. Nat. Struct. Biol..

[34] Löffler P, Schmitz S, Hupfeld E, Sterner R, Merkl R (2017). Rosetta:MSF: a modular framework for multi-state computational protein design. PLOS Comput. Biol..

[35] Frey KM, Georgiev I, Donald BR, Anderson AC (2010). Predicting resistance mutations using protein design algorithms. Proc. Natl Acad. Sci. USA.

[36] Reeve SM (2015). Protein design algorithms predict viable resistance to an experimental antifolate. Proc. Natl Acad. Sci. USA.

[37] Ojewole A (2017). OSPREY predicts resistance mutations using positive and negative computational protein design. Methods Mol. Biol. Clifton NJ.

[38] Roybal KT (2016). Engineering T cells with customized therapeutic response programs using synthetic notch receptors. Cell.

[39] Xie M, Fussenegger M (2018). Designing cell function: assembly of synthetic gene circuits for cell biology applications. Nat. Rev. Mol. Cell Biol..

[40] Di Stasi A (2011). Inducible apoptosis as a safety switch for adoptive cell therapy. N. Engl. J. Med..

[41] Caruso HG (2015). Tuning sensitivity of CAR to EGFR density limits recognition of normal tissue while maintaining potent antitumor activity. Cancer Res..

[42] Pegram HJ (2012). Tumor-targeted T cells modified to secrete IL-12 eradicate systemic tumors without need for prior conditioning. Blood.

[43] Shockley EM, Rouzer CA, Marnett LJ, Deeds EJ, Lopez CF (2019). Signal integration and information transfer in an allosterically regulated network. Npj Syst. Biol. Appl..

[44] DeRose R, Miyamoto T, Inoue T (2013). Manipulating signaling at will: chemically-inducible dimerization (CID) techniques resolve problems in cell biology. Pflüg. Arch. - Eur. J. Physiol..

[45] Gainza-Cirauqui P, Correia BE (2018). Computational protein design — the next generation tool to expand synthetic biology applications. Curr. Opin. Biotechnol..

[46] Silva, D.-A., Correia, B. E. & Procko, E. Motif-driven design of protein–protein interfaces. In *Computational Design of Ligand Binding Proteins* (ed. Stoddard, B. L.) vol. 1414, 285–304 (Springer New York, 2016). 10.1007/978-1-4939-3569-7_17 27094298

[47] Fleishman SJ (2011). RosettaScripts: a Scripting language interface to the Rosetta macromolecular modeling suite. PLoS ONE.

[48] Kuhlman B, Baker D (2000). Native protein sequences are close to optimal for their structures. Proc. Natl Acad. Sci. USA.

[49] Henikoff S, Henikoff JG (1992). Amino acid substitution matrices from protein blocks. Proc. Natl Acad. Sci. USA.

[50] Miller S, Janin J, Lesk AM, Chothia C (1987). Interior and surface of monomeric proteins. J. Mol. Biol..

[51] Kabsch W (2010). XDS. Acta Crystallogr. D. Biol. Crystallogr..

[52] McCoy AJ (2007). *Phaser* crystallographic software. J. Appl. Crystallogr..

[53] Emsley P, Lohkamp B, Scott WG, Cowtan K (2010). Features and development of Coot. Acta Crystallogr. D. Biol. Crystallogr..

[54] Adams PD (2010). *PHENIX*: a comprehensive Python-based system for macromolecular structure solution. Acta Crystallogr. D. Biol. Crystallogr..

