# Supplementary material for: A rational blueprint for the design of chemically-controlled protein switches
Source: Nat Commun. 2021 Oct 1;12:5754. doi: 10.1038/s41467-021-25735-9 (PMC8486872; doi:10.1038/s41467-021-25735-9)
Supplement: Supplementary file 1 — Supplementary Information [file 41467_2021_25735_MOESM1_ESM.pdf]

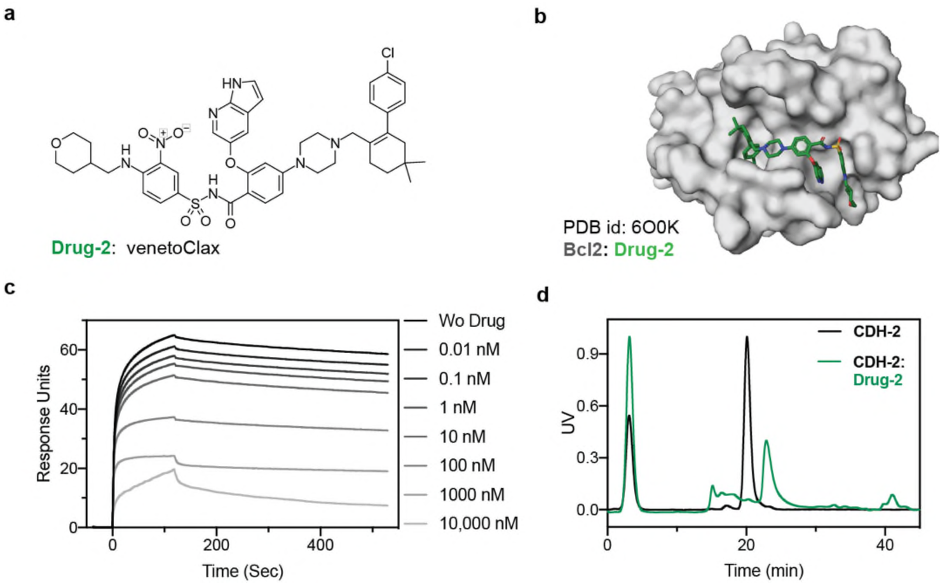

Supplementary Figure 1: Biophysical characterization of CDH-2.

a) Chemical structure of Venetoclax, used as CDH-2 disruptor (Drug-2). **b)** Crystal structure of Bcl2 (white surface) bound to Drug-2 (green sticks). **c)** Surface plasmon resonance drug competition assay. Kinetic curves of Drug-2 dissociating Bcl2:LD3 complex in SPR. Drug concentrations from 0.01 nM to 10 µM were pre-incubated with 4 µM LD3, then the mixtures were injected over a Bcl2-immobilized chip. Response units reflect the remaining interaction of Bcl2 and LD3 in the presence of serial diluted Drug-2. **d)** SEC-MALS analysis of CDH-2 showing Bcl2:LD3 complex with DMSO (black trace) and Bcl2:LD3 with Drug-2 did not result in complex formation and monomeric proteins (green trace), Bcl2 (19 kDa) and LD3 (16 kDa) eluted around 22 minutes.

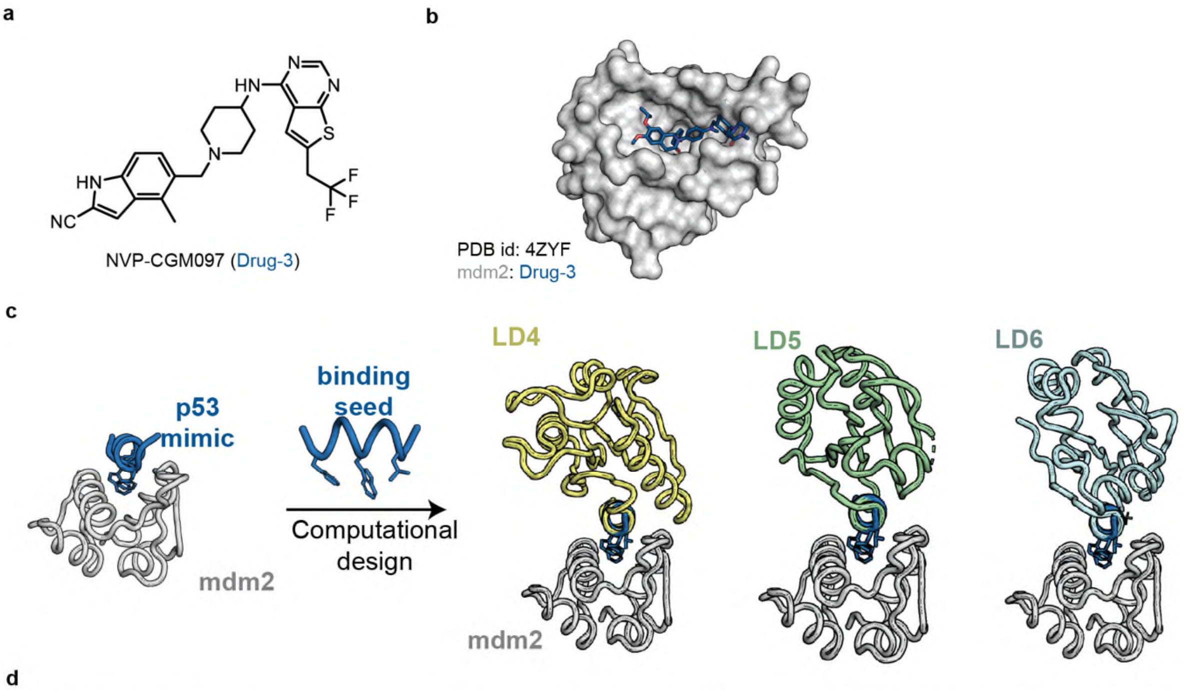

d

Design id	LD4	LD5	**LD6**
Scaffold PDB id	1AKU	2IFQ	2FWE
Scaffold protein name	Flavodoxin	Flavodoxin	DsbD
Organism of scaffold	*D. vulgaris*	*H. sapiens*	*M. musculus*
Rosetta ddG	-7.6	-8	-10
Globularity	1.04	0.98	0.93
VdW Dots to scaffold	980	660	670
SASA of seed	1769	1342	1337
Total # Mutations on scaffold	10	4	7

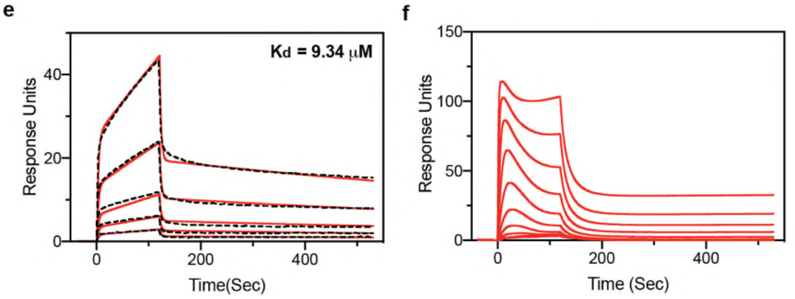

Supplementary Figure 2: Computational design and experimental testing of mdm2 binders.

a) Crystal structure of mdm2 in complex with its inhibitor:NVP-CGM097 (Drug-3). **b)** Chemical structure of Drug-3. **c)** p53 peptide was selected from the p53-mdm2 complex and shortened into an 8-residue motif which was matched against a database of > 11000 proteins using the MotifGraft protocol. Structures of three candidate designs (LD4-6) are shown in complex with mdm2. **d)** Table of designs and scores for the scoring/filtering criteria. Scaffold PDB ID: Protein Data Bank ID of the protein that was used as a scaffold to design each binder. Scaffold protein name: Name of the protein used as a scaffold. Organism of scaffold: Species origin of the native protein. Rosetta ddG: Computed delta-delta G interaction energy between designs and mdm2. Globularity: Globularity score for each designed

scaffold, where values closer to 1.0 are more globular. Globularity score was based on a metric created by Miller et al[1], further explained in Methods. vdW Dots to scaffold: Number of Van der Waals (VdW) contacts between the grafted motif and the scaffold. SASA of seed: buried surface area of the grafted motif in the scaffold. Total # mutations on scaffold: final number of residues in the scaffold that were mutated during the design process. **e-f)** Affinity measurements of mdm2 and designed binders: LD4 (e), LD5 (f). Designed binders at concentrations from 125 to 2000 nM with 2-fold dilutions were injected over mdm2 immobilized chips. Black dashed curves represent the sensorgrams and the fitting curves are in solid red curves. K_ds were computed using a 1:1 binding model.

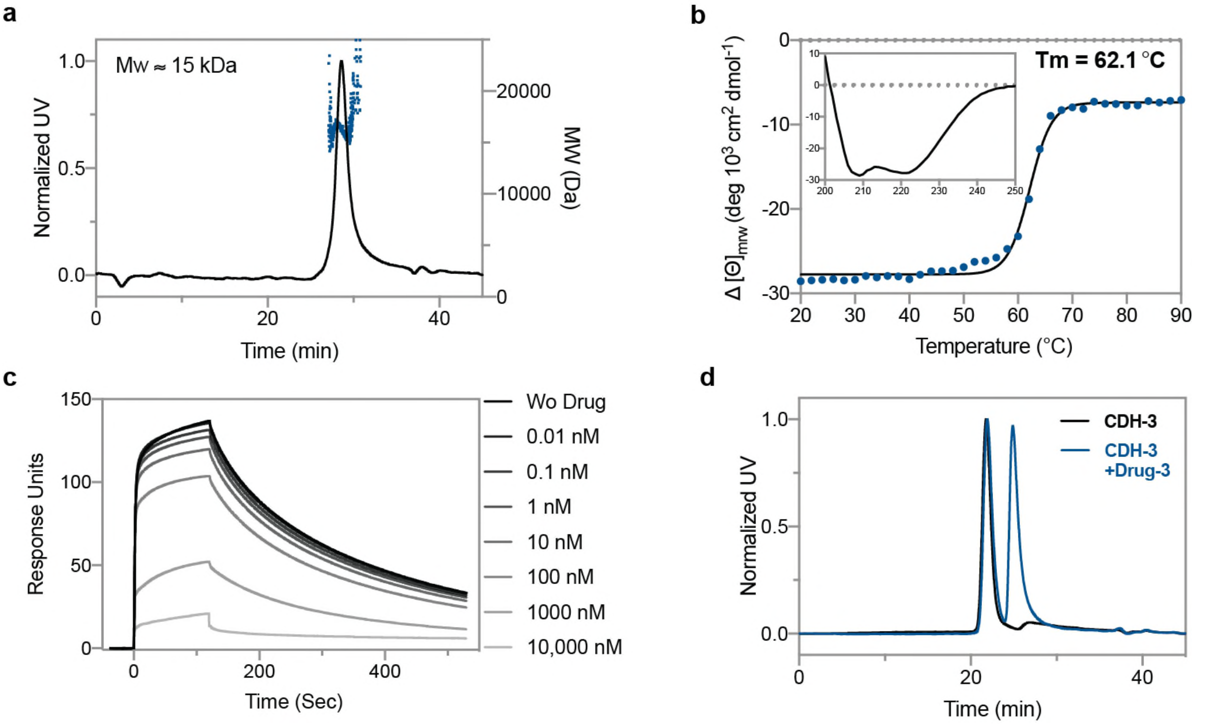

Supplementary Figure 3: Biophysical characterization of CDH-3.

a) SEC-MALS analysis showed that LD6 is a monomeric protein with a molecular weight around 15 kDa. **b)** Thermostability of LD6. Circular Dichroism spectrum showed a melting temperature of 62 ˚C and a typical helical absorption curve at 220 nm. **c)** Surface plasmon resonance drug competition assay. Kinetic curves of Drug-3 dissociating mdm2:LD6 complex in SPR. Drug concentrations from 0.01 nM to 10 µM were pre-incubated with 4 µM LD6, then the mixtures were injected over an mdm2-immobilized chip. Response units reflect the remaining interaction of mdm2 and LD6 in the presence of serially diluted Drug-3. **d)** SEC-MALS analysis of CDH-3 showing mdm2:LD6 complex with DMSO (black trace), while dissociation of the mdm2:LD6 with Drug-3 could be observed and monomeric proteins (blue trace), mdm2 (11 kDa) and LD6 (15 kDa) eluted around 25 minutes.

a

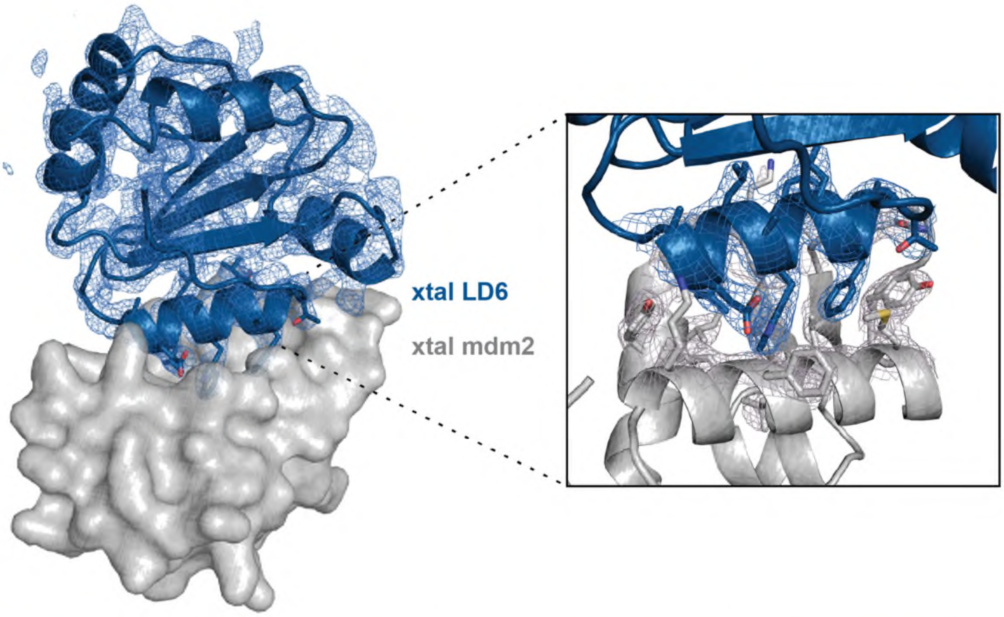

b

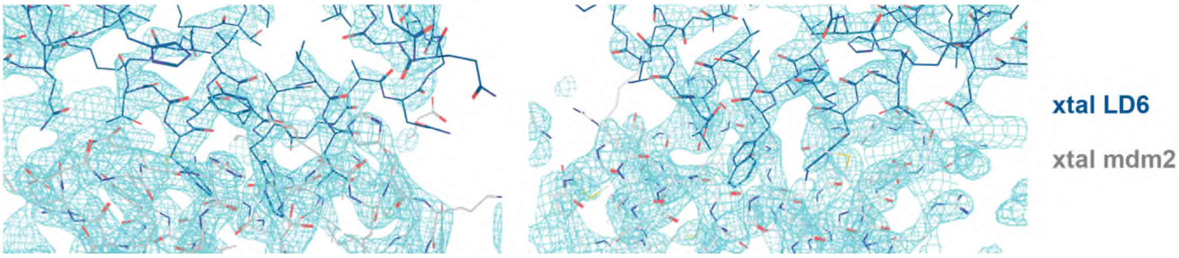

Supplementary Figure 4: 2mFo-mFc electron density and stereo maps of the mdm2:LD6 crystal structure.

a) 2mFo-mFc electron density maps of CDH-3 complex. Maps are contoured at 1 σ and carved around the structure at 1.6 Å. Side chains are shown in sticks representation. **b)** Stereo representations of electron density maps of CDH-3 complex.

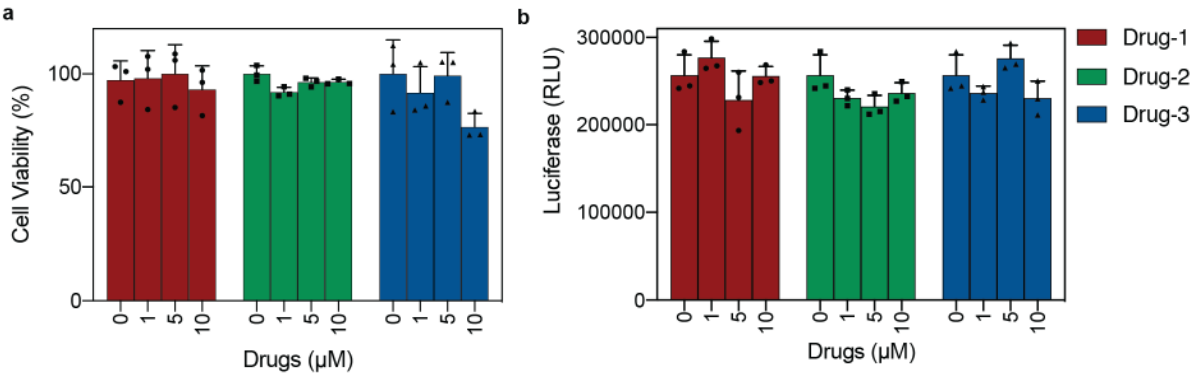

Supplementary Figure 5: Drug toxicity assessment.

a) Drug effects on cell viability of HEK293T cells. HEK293T cells were added different concentrations of drugs for an incubation period of 24 hours. WST-8 was added 24 h after drug incubation and the absorbance at 450 nM was measured 4 h later. Absorbance values were normalized to the positive control of DMSO groups (0 μM), each bar represents the mean of three biological replicates ± s.d, overlaid with a scatter dot plot of the original data points. **b)** Effect of drugs on the protein production of HEK293T cells. The HEK293T cells were transfected with the Luciferase reporter and constitutive Gal4-Rel65 expression plasmid (pCMV-Gal4-Rel65-pA). Drugs were added to the transfected cells 24 hours post-transfection, quantification of luciferase activity 24 hours after the addition of the drugs. Each bar represents the mean of three biological replicates ± s.d overlaid with a scatter dot plot of the original data points.

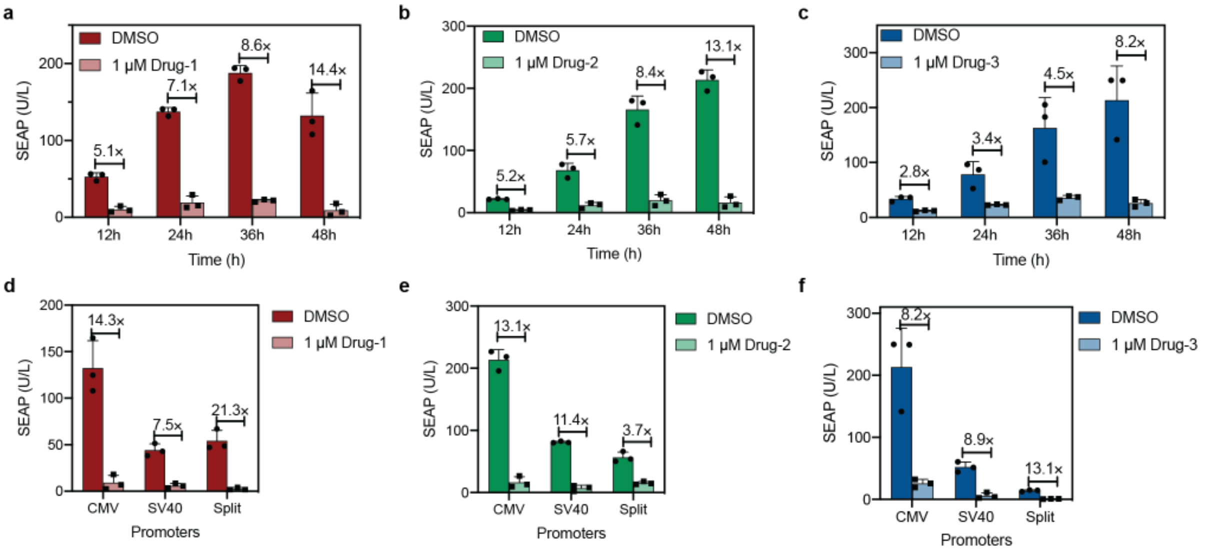

Supplementary Figure 6: Optimization of CDH(1-3)-TFs dynamic response ranges.

a-c) CDH-TFs fold-change activity between drug treated (1 µM) and untreated (DMSO) conditions, showing the quantification of SEAP expression after 12, 24, 36 and 48 h drug treatment. HEK293T cells were transfected with SEAP reporter (S132) and respective CDH-(1-3)-TFs, and Drug-(1-3) were replenished every 12 hours. Culture medium at time points of 12, 24, 36 and 48 h were collected and SEAP activity measured. **d-f)** Comparison of different promoters on the effects of CDHs-TFs fold-change activity between drug treated (1 µM) and untreated (DMSO) conditions. HEK293T cells were transfected with CMV (original CDH-(1-3)-TFs which was driven by CMV expression), SV40 (CDH-TF (Gal4-CDHprotein1-P2A-CDHprotein2-p65) driven by SV40 promoters), and Split (two plasmids encoding Gal4-CDHprotein1 and CDHprotein2-p65 under SV40 promoters). Quantification of SEAP expression was assessed after 48 hours drug treatment. a-f) The bar charts show the mean ± s.d. of $n = 3$ biologically independent samples overlaid with a scatter dot plot of the original data points.

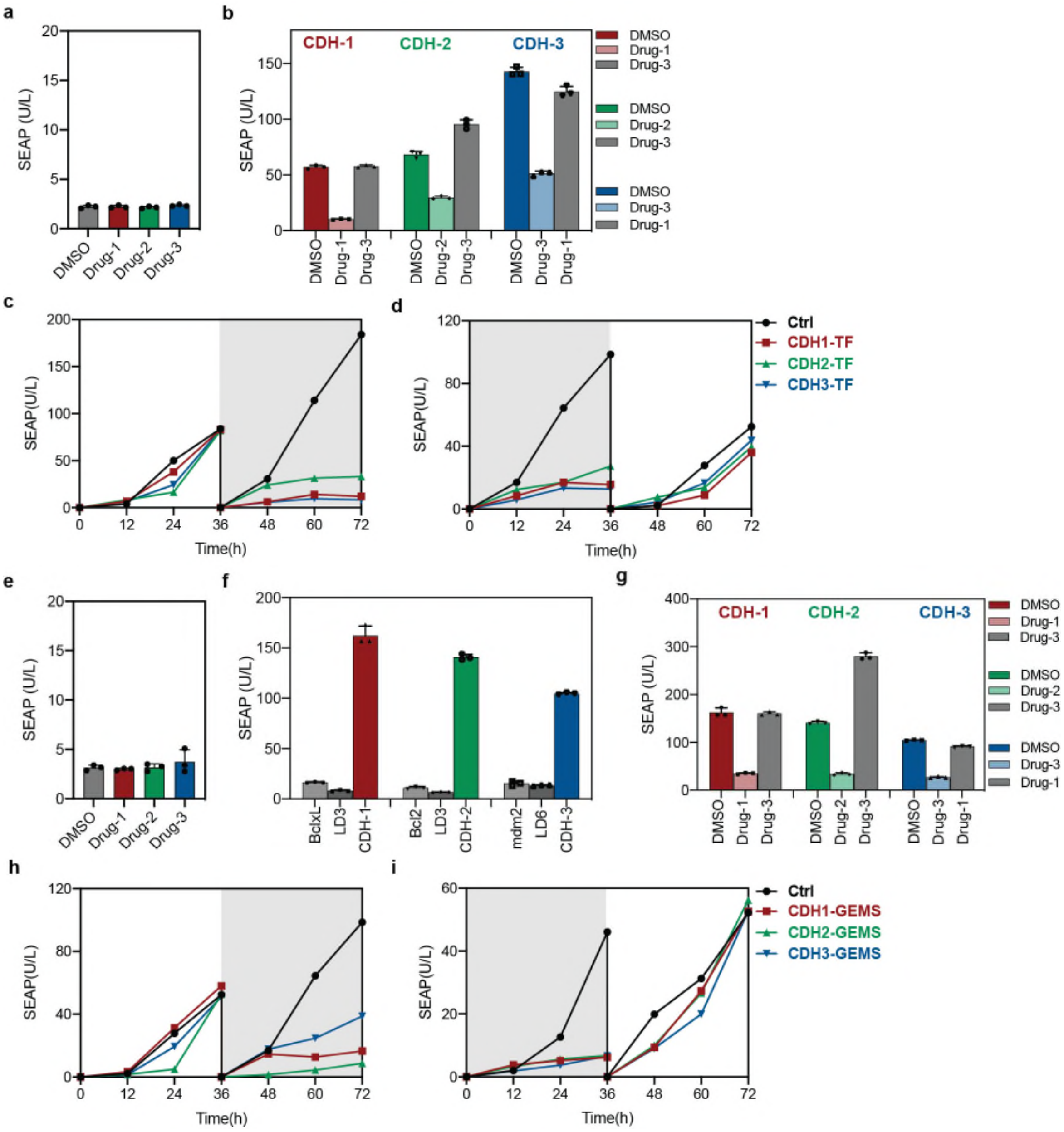

Supplementary Figure 7: Dose-dependence and reversibility characterization of CDHs in CDH-TF and CDH-GEMS systems.

a) Background signal of SEAP reporter in the absence of the CDH-TF system. HEK239T cells were transfected with SEAP reporter S132 and treated with 1 µM Drugs 12 hours post transfection. Quantification of SEAP was performed 24 hours after drug treatment. **b)** Drug specificity of CDH-TFs. Quantification of SEAP activity of three CDHs with DMSO (negative control; dark color), specific (designed drug induced OFF switches; light color) and unspecific drug treatment (testing for possible unintended drug induced effects; gray). **c)** Dynamic regulation of SEAP expression mediated by CDHs-TF in modes of ON-OFF. The control samples (Ctrl) were transfected with the SEAP reporter S132 and constitutive Gal4-Rel65 expression plasmid S108 (pCMV-Gal4-Rel65-pA). During the OFF-time period shown in gray box, CDH-TF cells were cultured with 500 nM of the corresponding drugs while Ctrl cells were treated with same concentration of DMSO, drugs/DMSO were replenished every 12 hours at time points of 36h, 48h and 60h. Cells were split at 36h changing medium to with/without

drugs respectively. **d)** Dynamic regulation of SEAP expression mediated by CDHs-TF in modes of OFF-ON. Time point 0 was set 12 hours post transfection to start the intermittent drug treatment shown in gray boxes, CDH-TF cells were cultured with 500 nM of the corresponding drugs while Ctrl cells were treated with same concentration of DMSO. Drugs/DMSO were refreshed at time points of 0h, 12h, 24h. Cells were split at 36h changing medium to without/with drugs respectively. **e)** Background signal of SEAP reporter in the absence of the CDH-GEMS system. HEK293T cells were transfected with pLS566 SEAP reporter plasmids and treated with drugs. Quantification of SEAP was performed 24 hours post drug treatment. **f)** SEAP activity upon transfecting single or paired constructs of CDH-GEMS. Single constructs in gray remain inactive, while paired transfection of CDHs (1-red, 2-green and 3-blue) activate SEAP expression. **g)** Drug specificity test on CDH-GEMS. Quantification of SEAP activity of three CDHs with DMSO (dark color), specific (light color) and unspecific drug treatment (gray). Samples of CDHs (1-red, 2-green and 3-blue) were treated with their specific disruptors in faded color labelled as Drug-1, Drug-2 and Drug-3, and the unspecific Drug-3 used for CDH-1 and CDH-2, Drug-1 as the unspecific disruptor for CDH-3. **h-i)** Dynamic regulation of SEAP expression mediated by CDH-GEMS. Positive control was transfected with CDH-1-GEMS and with DMSO treatment. Experiments were done with the same principle in panel b and c. Samples (culture medium) in panels of b, c, f and g were collected every 12 hours. Values were normalized to zero based on the SEAP expression at time 0. a, b, e, f, g) The bar charts show the mean ± s.d. of $n = 3$ biologically independent samples overlaid with a scatter dot plot of the original data points.

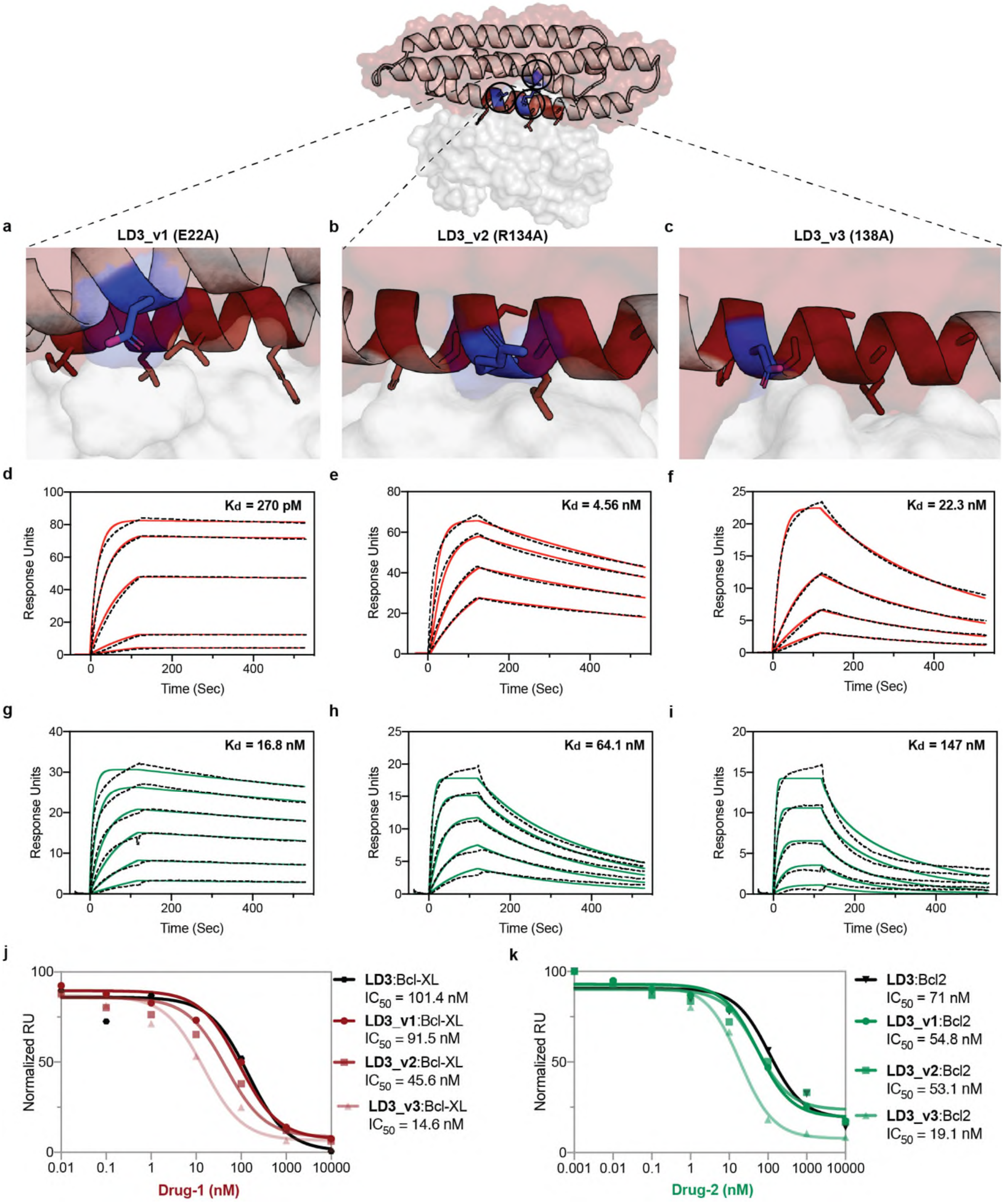

Supplementary Figure 8: Evaluation of weak affinity variants of the LD3:BclxL and LD3:Bcl2 complexes.

a-c) Alanine mutations of LD3_v1, LD3_v2 and LD3_v3. The LD3 protein (light red cartoon and surface) is shown in complex with Bcl-XL protein (white surface). A 12-amino acid motif from the BiM BH3 peptide (dark red cartoon) is highlighted with hotspot residues shown in red sticks and with each residue mutated to alanine shown in blue sticks. **d-f)** Affinity measurements of Bcl-XL and LD3 (v1_v3) by SPR. Indicated concentrations of LD3 mutants were injected over the Bcl-XL immobilized chips. The binding

sensorgrams (black dashed curves) are plotted with fitted curves (solid red). **g-i)** Affinity measurements of Bcl2 and LD3 (v1_v3) by SPR. Indicated concentrations of LD3 mutants were injected over the Bcl2 immobilized chips. The binding sensorgrams (black dashed curves) are plotted with fitted curves (solid green). **d-i)** K_d was calculated by a 1:1 binding model. **j-k)** SPR IC_{50} determinations of LD3 mutants with Bcl-XL (**j**) and Bcl2 (**k**). Indicated concentrations of Drug-1 or Drug-2 were premixed with 4 µM LD3 and mutants, then injected into Bcl-XL and Bcl2 immobilized chips, respectively. Readouts from the sensorgrams were extracted at the time point 120 s to calculate the IC_{50}s using a three-parameter nonlinear regression.

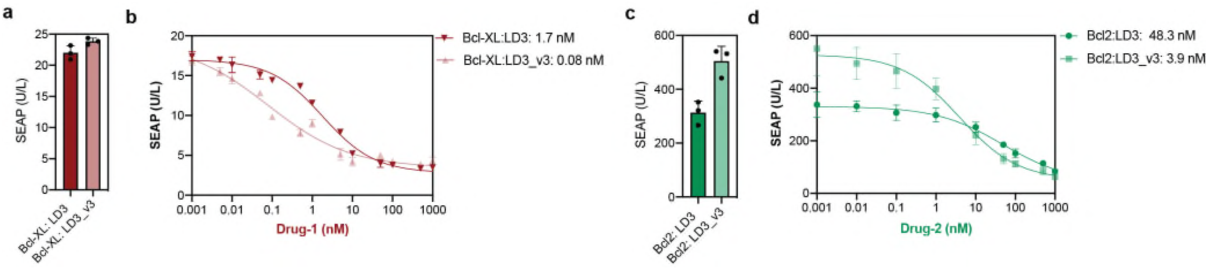

Supplementary Figure 9: Comparison of ON-state reporter expression of weak affinity variants of LD3 in CDH-1/2-GEMS platform.

a) ON-state expression of CDH-1-GEMS with LD3 and LD3_v3. **b)** Drug dose-dependent responses in engineered cells determined for the CDH-1-GEMS with LD3 and LD3_v3. **c)** ON-state expression of CDH-2-GEMS with LD3 and LD3_v3. **d)** Drug dose-dependent responses in engineered cells determined for the CDH-2-GEMS with LD3 and LD3_v3. In the data shown HEK293T cells were transfected with corresponding CDH-GEMS pairs, treated with drugs ranging from 0.001 to 1 µM. SEAP quantification was done 24 hours after drug treatment. a, c) The bar charts show the mean ± s.d. of *n* = 3 biologically independent samples overlaid with a scatter dot plot of the original data points. b, d) Each data point represents the mean of n = 3 biological replicates, and the IC_{50}s were calculated using four-parameter nonlinear regression.

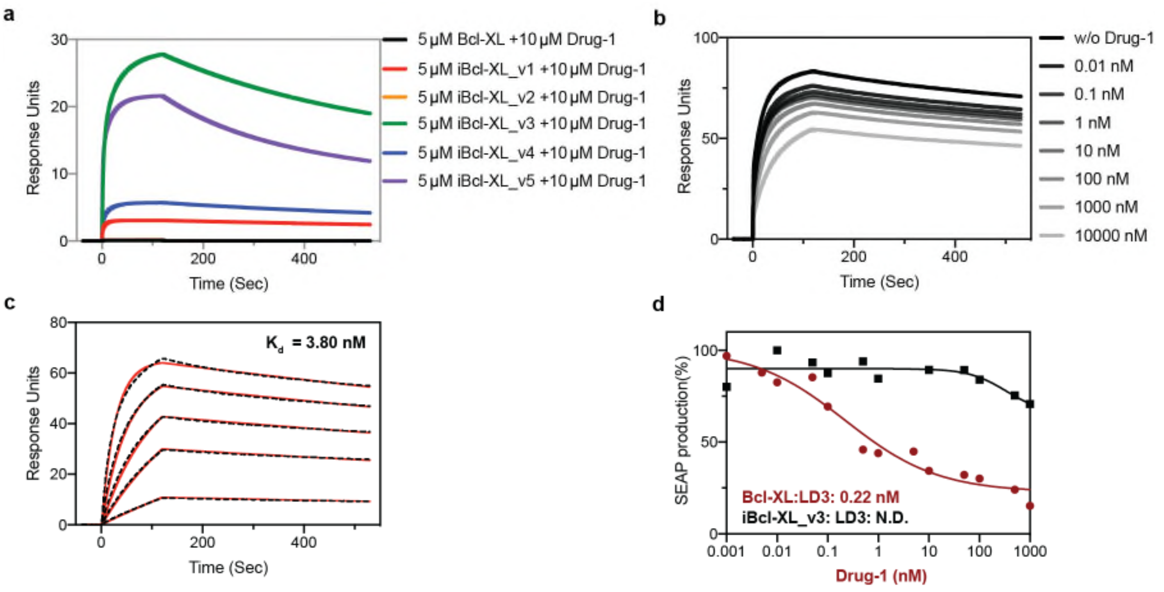

Supplementary Figure 10: Biophysical and cellular characterization of designed Drug-1-resistant BclxL mutants.

a) Pre-screening of Drug-1 resistant Bcl-XL mutants by SPR. 5 µM of Bcl-XL and five mutants were mixed with 10 µM of Drug-1 and injected over the LD3 immobilized chip to analyse the binding response. Drug-1 resistant mutants showed higher response. Drug-1 showed complete inhibition to Bcl-XL (Black) and iBcl-XL_v2 (orange; not visible in the graph due to overlay with Bcl-XL), significant inhibition to iBcl-XL_v1 (red) and iBcl-XL_v4 (blue), mild inhibition to iBcl-XL_v5 (purple) and the weakest inhibition to iBcl-XL_v3 (green). **b)** SPR competition assay of Drug-1 dissociating iBcl-XL_v3:LD3 complex. Serial dilutions of Drug-1 were pre-mixed with 4 µM LD3 and injected over iBcl-XL_v3 immobilized chip to collect the response. **c)** Dissociation constant measurement of iBcl-XL_v3 and LD3. Sensorgrams are in black dashed curves and the fitted curves in solid red lines. K_d was using a 1:1 binding model. **d)** IC_{50}s of Drug-1 dissociating Bcl-XL:LD3 versus iBcl-XL_v3:LD3 in the CDH-GEMS system. The plot shows the dose response of 24 hours after the addition of serial Drug-1 concentrations. Values were normalized to the positive control (DMSO group). Each data point represents the mean of n = 3 biological replicates, and the IC_{50}s were calculated using four-parameter nonlinear regression.

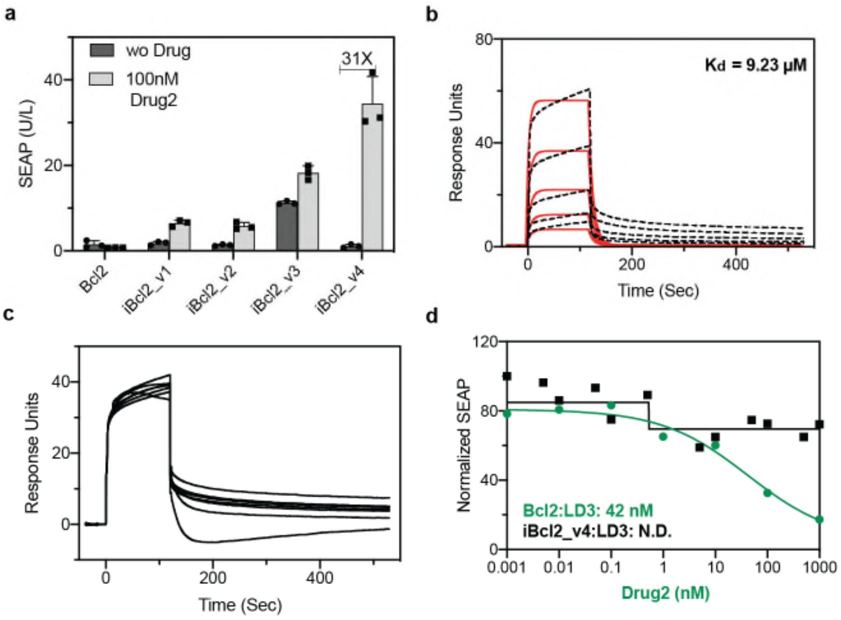

Supplementary Figure 11: Cellular and biophysical characterization of Drug-2 resistant Bcl2 mutants.

a) Pre-screening of Drug-2 resistant Bcl2 mutants. Bcl2 mutants were cloned into the AIR-GEMS platform and co-transfected with P_{SV40}-IgK-Bcl2-$GGGGS_{X3}$-LD3-EpoRm-IL-6RBm-pA to test ON-switch behavior. P_{SV40}-IgK-Bcl2-EpoRm-IL-6RBm-pA was used as the negative control, and all groups were exposed to 100nM DMSO (dark grey) versus Drug-2 (light grey). Each bar represents the mean of three biological replicates ± s.d, overlaid with a scatter dot plot of the original data points. **b)** Dissociation constant measurement of iBcl2_v4 and LD3 in SPR. Sensorgrams are in black dashed curves and the fitted curves in solid red lines. K_D was calculated by a 1:1 binding model in the biacore system. **c)** Drug competition assay measured Drug-2 disrupting iBcl2_v4:LD3 complex. Serial dilutions of Drug-2 were mixed with 4 µM LD3 and injected over iBcl2_v4 immobilized chip to collect the response. **d)** IC_{50}s of Drug-2 disrupting Bcl2:LD3 and iBcl2_v4:LD3 in the CDH-GEMS system. Dose response of diluted Drug-2, 24 hours after treatment. Each data point represents the mean of n = 3 biological replicates, and the IC_{50}s were calculated using four-parameter nonlinear regression.

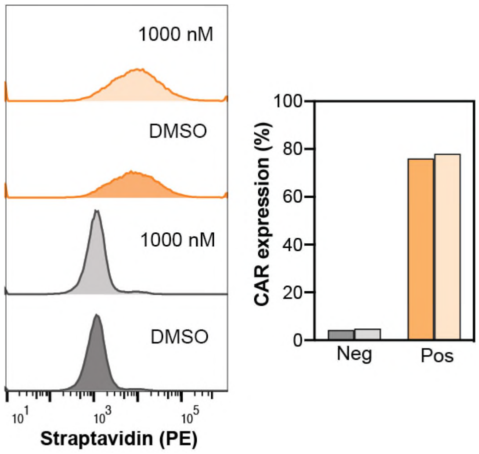

Supplementary Figure 12: Control experiments for the AIR-TF CAR expression system.

Anti-HER2 CAR staining showed in flow cytometry plots and overall quantified percentages. Drug-1 does not induce HER2 CAR expression in the absence of the CDH-TF (curves in gray labeled Neg) and does not alter the expression in a constitutive system with a fused Gal4-p65 construct (curves in orange labeled Pos). Cells were treated with 1 μM Drug-1 or equivalent concentration of DMSO 24 hours after transfection, and anti-HER2 CAR expression were assessed 24 hours after drug treatment.

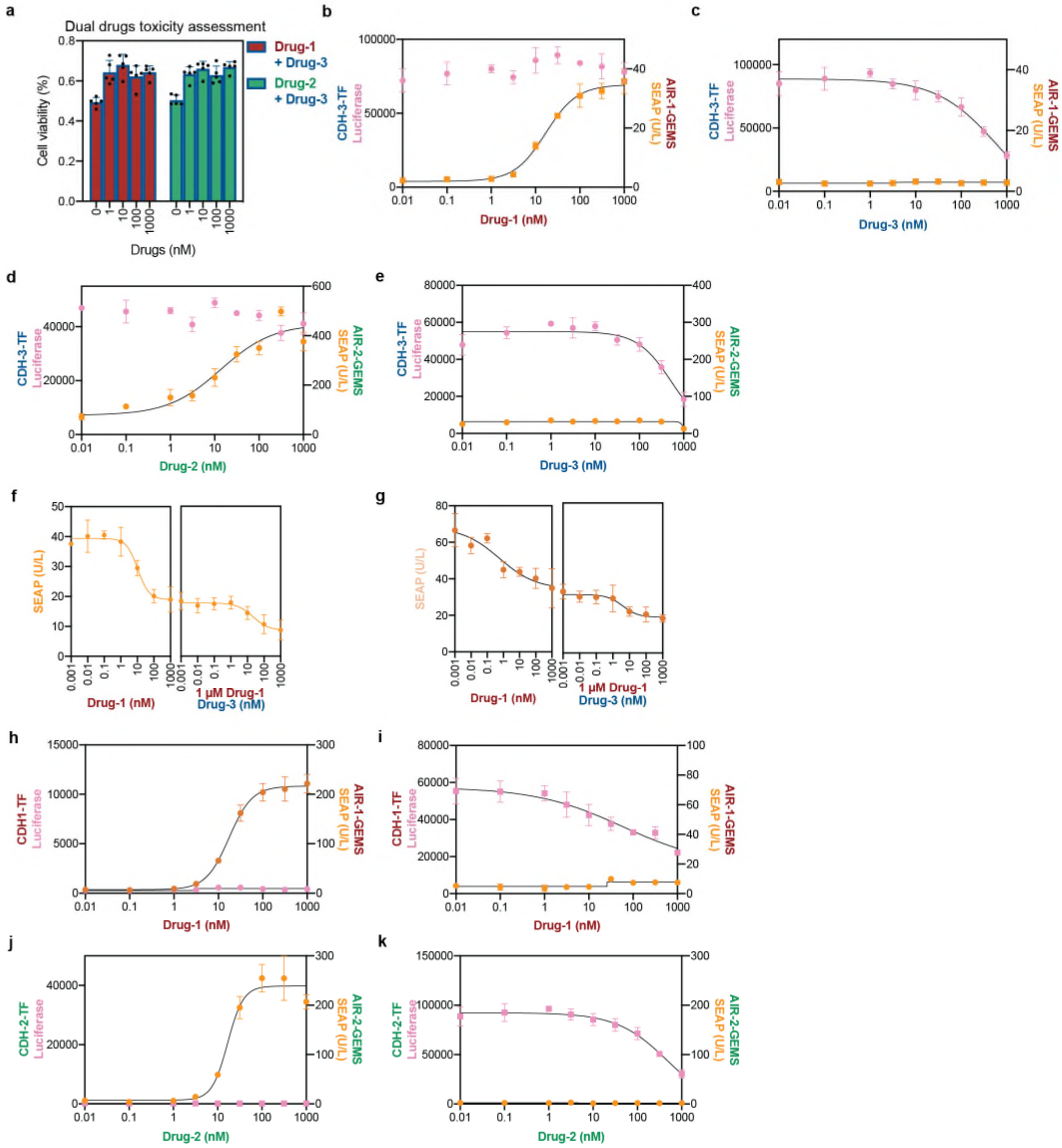

Supplementary Figure 13: Orthogonal chemical switches enable implementation of multi-input multi-output control modes in mammalian cells.

a) Dual drug toxicity tests in 293T cells. Drug-1 + Drug-3 and Drug-2 + Drug-3 were diluted to the concentrations of 1 nM, 10 nM, 100 nM and 1000 nM each drug in cell culture medium. CCK8 assay was performed 24 hours after drug treatment. Each bar represents the mean of three biological replicates ± s.d, overlaid with a scatter dot plot of the original data points. **b-c)** Cells were co-transfected with AIR-1-GEMS and CDH-3-TF regulate SEAP and Luciferase expression, respectively. Drug concentrations ranged from 0.001 nM to 1 μM and different combinations were added depending on the protein components, Drug-1 only (b) and Drug-3 only (c). **d-e)** Cells were co-transfected with AIR-2-GEMS and CDH-3-TF regulate SEAP and Luciferase expression, respectively. Drug concentrations ranged from 0.001 nM to 1 μM and different combinations were added depending on the protein

components, Drug-2 only (d) and Drug-3 only (e). **f)** Quantification of SEAP activity controlled by CDH-1-TF and CDH-3-TF circuits. Drug-1 concentrations ranged from 0.001 nM to 1 µM (left) and 1 µM Drug-1 with serial diluted Drug-3 from 0.001 nM to 1 µM (right). **g)** Quantification of SEAP activity controlled by CDH-1-GEMS and CDH-3-GEMS circuits. Drug-1 concentrations ranged from 0.001 nM to 1 µM (left) and Drug-3 dilution along with 1 µM Drug-1 (right). **h-i)** Quantification of SEAP and Luciferase activities under Drug-1. AIR-1-GEMS coupled with SEAP expression circuits (i) and CDH-1-TF circuits which control the Luciferase production (j). **j-k)** Quantification of SEAP and Luciferase activities under Drug-2. AIR-2-GEMS coupled with SEAP expression circuits (k) and CDH-2-TF circuits which control the Luciferase production (l). b-k) Each data point represents the mean of n = 3 biological replicates, and the dose-dependent curve were fitted using four-parameter nonlinear regression.

Supplementary Table 1: Crystal structure information of mdm2: LD6

	**LD6/mdm2**
**Data collection**	
Space group	P 4_3 2 2
Cell dimensions	
a, *b*, *c* (Å)	73.2, 73.2, 92.2
α, β, γ (°)	90.0, 90.0, 90.0
Resolution (Å)	45.16 – 2.95 (3.13– 2.95)*
$I / \sigma I$	22.2 (2.2)
Completeness (%)	99.8 (99.8)
Redundancy	6.9 (7.4)
R_{meas}	0.07 (0.84)
CC1/2**	0.99 (0.67)
**Refinement**	
No. reflections	5,646
R_{work} / R_{free}	0.20 / 0.25
No. atoms	1,656
Protein	1,656
B-factors	
Protein	86.1
R.m.s. deviations	
Bond lengths (Å)	0.008
Bond angles (°)	1.61
**PDB code**	**7AYE**

*Values in parentheses are for highest-resolution shell.

**CC1/2 refers to Pearson's correlation coefficients (CC) between intensity estimates from half data sets.

Supplementary Table 2: Prediction of Drug-1 resistant Bcl-XL mutants.

Mutations: Specific mutations modeled from wildtype Bcl-XL. Positive_Bound: Score of the LD3:iBcl-XL mutant complex.

Positive_unbound: Score of the iBcl-XL mutant in its unbound (apo) state.

Negative_Bound: score of the Drug-1:iBcl-XL complex.

Negative_unbound: Score of iBcl-XL mutant in its unbound (apo) state, based on the crystal structure of Drug-1:Bcl-XL.

Ratio_unbound: (Positive_bound - Positive_unbound) - (Negative_bound - Negative_unbound).

Ratio_Bound: (Positive_bound - Negative_bound)

Name	Mutations	Positive_Bound	Positive_unbound	Negative_Bound	Negative_unbound	Ratio_unbound	Ratio_Bound
iBcl-XL_v1	T109L, A149L	-362.525	-164.568	-130.467	-155.829	-223.319	-232.058
iBcl-XL_v2	A149V	-366.699	-139.013	-180.803	-150.961	-197.597	-185.649
iBcl-XL_v3	R102E, F105I	-365.076	-161.623	-170.317	-149.164	-182.3	-194.759
iBcl-XL_v4	R102F, T109V	-368.015	-164.292	-174.012	-146.991	-176.702	-194.003
iBcl-XL_v5	E98S, F105I	-371.963	-160.491	-172.324	-148.471	-187.619	-199.639

Supplementary Table 3: Prediction of Drug-2 resistant Bcl2 mutants.

Mutations: Specific mutations modeled from wildtype Bcl2. Positive_Bound: Score of the LD3:iBcl2 mutant complex.

Positive_unbound: Score of the iBcl2 mutant in its unbound (apo) state.

Negative_Bound: score of the Drug-2:iBcl2 complex.

Negative_unbound: Score of iBcl2 mutant in its unbound (apo) state, based on the crystal structure of Drug-2:Bcl2.

Ratio_unbound: (Positive_bound - Positive_unbound) - (Negative_bound - Negative_unbound).

Ratio_Bound: (Positive_bound - Negative_bound)

Three additional mutations were enriched in the top designs, and therefore we designed a further version, iBcl2_v4 (100V_103N_202H).

Name	Mutations	Positive_Bound	Positive_unbound	Negative_Bound	Negative_unbound	Ratio_unbound	Ratio_Bound
iBcl2_v1	V156I_Y202H	-383.197	-207.19	-223.495	-207.19	-159.702	-159.702
iBcl2_v2	D103N_Y202H	-373.831	-193.74	-222.264	-193.74	-151.567	-151.567
iBcl2_v3	A100T_D103S	-371.663	-191.485	-220.949	-191.485	-150.714	-150.714
iBcl2_v4	100V_103N_202H						

Supplementary Table 4: Protein sequences of CDHs(1-3).

Name	Sequences
CDH-1-Bcl-XL	MSQSNRELVVDFLSYKLSQKGYSWSQFSDVEENRTEAPEGTESEAVKQALREAGDEFELRYRRAFSDLTSQLHITPGTAYQSFEQVVNELFRDGV<br>NWGRIVAFFSFGGALCVESVDKEMQVLVSRIAAWMATYLNDHLEPWIQENGGWDTFVELYGNNAAAESRKGQER
CDH-2-Bcl2	MAHPGRTGYDNREIVMKYIHYKLSQRGYEWDAGDDVEENRTEAPEGTESEVVHLTLRQAGDDFSRRYRRDFAEMSSQLHLTPFTARGRFATVVEE<br>LFRDGVNWGRIVAFFEFGGVMCVESVNREMSPLVDNIALWMTEYLNRHLHTWIQDNGGWDAFVELYGPSMR
CDH-1/2-LD3	QRWELALGRFLEYLSWVSTLSEQVQEELLSSQVTQELRALMDETMKELKAYKSELEEQLTPVAEETRARLSKELQAAQARLGADMEDVRGRLVQY<br>RGEVQAMLGQSTEELRVRLASHLIALALRLIGDAFDLQKRLAVY
CDH-3-mdm2	GPLGSSQIPASEQETLVRPKPLLLKLLKSVGAQKDTYTMKEVLFYLGQYIMTKRLYDAAQQHIVYCSNDLLGDLFGVPSFSVKEHRKIYTMIYRN<br>LV
CDH-3-LD6	HLNFTQIKTAFALYWALLEAQGKPVMLDLYADWCVACKEFEKYTFSDPQVQKALADTVLLQANVTANDAQDVALLKHLNVLGLPTILFFDGQGQE<br>HPQARVTGFMDAETFSAHLRDRQPHHH

Supplementary Table 5: LD3 variants with low affinity and drug-insensitive receptors.

Name	Sequences
LD3_v1	QRWELALGRFL**A**YLSWVSTLSEQVQEELLSSQVTQELRALMDETMKELKAYKSELEEQLTPVAEETRARLSKELQAAQARLGADMEDVRGRLVQYRGEVQAMLGQSTEELRVRLASHLIALALRLIGDAFDLQKRLAVY
LD3_v2	QRWELALGRFLEYLSWVSTLSEQVQEELLSSQVTQELRALMDETMKELKAYKSELEEQLTPVAEETRARLSKELQAAQARLGADMEDVRGRLVQYRGEVQAMLGQSTEELRVRLASHLIALAL**A**LIGDAFDLQKRLAVY
LD3_v3	QRWELALGRFLEYLSWVSTLSEQVQEELLSSQVTQELRALMDETMKELKAYKSELEEQLTPVAEETRARLSKELQAAQARLGADMEDVRGRLVQYRGEVQAMLGQSTEELRVRLASHLIALALRLIG**A**AFDLQKRLAVY
iBcl-XL_v3	MSQSNRELVVDFLSYKLSQKGYSWSQFSDVEENRTEAPEGTESEAVKQALREAGDEFELRY**E**RA**I**SDLTSQLHITPGTAYQSFEQVVNELFRDGVNWGRIVAFFSFGGALCVESVDKEMQVLVSRIAAWMATYLNDHLEPWIQENGGWDTFVELYGNNAAAESRKGQER
iBcl2_v4	MAHPGRTGYDNREIVMKYIHYKLSQRGYEWDAGDDVEENRTEAPEGTESEVVHLTLRQ**V**GD**N**FSRRYRRDFAEMSSQLHLTPFTARGRFATVVEELFRDGVNWGRIVAFFEFGGVMCVESVNREMSPLVDNIALWMTEYLNRHLHTWIQDNGGWDAFVEL**H**GPSMR

Supplementary Table 6: Plasmids in cellular applications

Plasmid	Description and cloning strategy	Reference
pPKm-118	P_{UAS}-driven vector expressing reporter gene Luciferase (P_{5XUAS}-Luciferase-pA).	Addgene #90491
S132	P_{UAS}-driven vector expressing reporter gene secreted embryonic alkaline phosphatase (P_{5XUAS}-SEAP-pA).	This work
S108	Constitutive P_{hCMV}-driven mammalian expression vector (P_{hCMV}-Gal4-Rel65-pA), gene cloned from plasmid: pCS2+ Gal4-GBP2-IRES-GBP7-p65	Addgene #50020
S111	Constitutive P_{hCMV}-driven mammalian expression vector of CDH-1-TF cassette (P_{hCMV}-Gal4-Bcl-XL-P2A-LD3-Rel65-pA), cloned into the NheI digested S108 by Gibson assembly	This work
S112	Constitutive P_{hCMV}-driven mammalian expression vector of CDH-2-TF cassette (P_{hCMV}-Gal4-Bcl2-P2A-LD3-Rel65-pA), cloned into the NheI digested S108 by Gibson assembly	This work
S113	Constitutive P_{hCMV}-driven mammalian expression vector of CDH-3-TF cassette (P_{hCMV}-Gal4-mdm2-P2A-LD6-Rel65-pA), cloned into the NheI digested S108 by Gibson assembly	This work
pLS13	Mammalian reporter plasmid for STAT3-induced SEAP expression (OStat3-$P_{hCMVmin}$-SEAP-pA)	This work
pLS15	Constitutive P_{hCMV}-driven mammalian STAT3 expression vector (P_{hCMV}-STAT3-pA)	This work
S184	Mammalian CDH-1-GEMS expression vector (P_{SV40}-IgK-Bcl-XL-EpoRm-IL-6RBm-pA), original plasmid was from pLeo619 by changing the extracellular interaction domain into Bcl-XL to form CDH-1 with LD3(S185), cloned into the SpeI digested pLEo619 by Gibson assembly with the secrection signal of IgK	This work
S193	Mammalian CDH-2-GEMS expression vector (P_{SV40}-IgK-Bcl2-EpoRm-IL-6RBm-pA), co-transfection with S185 to form the full CDH-2-GEMS machinery	This work
S185	Mammalian expression vector of binder protein LD3, used together with S184 and S193 to form CDH-1 and CDH-2 respectively (P_{SV40}-IgK-LD3-EpoRm-IL-6RBm-pA)	This work
S189	Mammalian CDH-3-GEMS expression vector (P_{SV40}-IgK-mdm2-EpoRm-IL-6RBm-pA), work with S190	This work
S190	Mammalian CDH-3-GEMS expression vector (P_{SV40}-IgK-LD6-EpoRm-IL-6RBm-pA), work with S189	This work
S215	Mammalian AIR-1-GEMS expression vector (P_{SV40}-IgK-Bcl-XL-$GGGGS_{X3}$-LD3-EpoRm-IL-6RBm-pA), two domains of CDH-1 were genetically fused by the three repeats of GS linker and cloned into EpoR-IL-6RB receptor plasmid	11

Plasmid	Description and cloning strategy	Reference
S226	Mammalian AIR-1-GEMS expression vector (P_{SV40}-IgK-iBcl-XL_v3-EpoRm-IL-6RBm-pA), the drug insensitive Bcl-XL variant, Bcl-XLMut3 was cloned into EpoR-IL-6RB receptor backbone and co-worked with S215 to form the AIR-1-GEMS switch	This work
S222	Mammalian AIR-2-GEMS expression vector (P_{SV40}-IgK-Bcl2-$GGGGS_{X3}$-LD3-EpoRm-IL-6RBm-pA), two domains of CDH-1 were genetically fused by the three repeats of GS linker and cloned into EpoR-IL-6RB receptor plasmid	This work
S228	Mammalian AIR-1-GEMS expression vector (P_{SV40}-IgK-iBcl2_v4-EpoRm-IL-6RBm-pA), the drug insensitive Bcl2 variant, Bcl2Mut7 was cloned into EpoR-IL-6RB receptor backbone and co-worked with S222 to form the AIR-2-GEMS switch	This work
S133	Constitutive P_{hSV40}-driven mammalian expression vector of CDH-1-TF cassette (P_{hSV40}-Gal4-Bcl-XL-P2A-LD3-Rel65-pA), SV40 promoter driven CDH-1-TF	This work
S134	Constitutive P_{hSV40}-driven mammalian expression vector of CDH-2-TF cassette (P_{hSV40}-Gal4-Bcl-2-P2A-LD3-Rel65-pA), SV40 promoter driven CDH-2-TF	This work
S135	Constitutive P_{hSV40}-driven mammalian expression vector of CDH-3-TF cassette (P_{hSV40}-Gal4-mdm2-P2A-LD6-Rel65-pA), SV40 promoter driven CDH-3-TF	This work
S333	Constitutive P_{hSV40}-driven mammalian expression vector of Gal4-Bcl-XL cassette (P_{hSV40}-Gal4-Bcl-XL-pA), Co-express with S335 to form the split system of CDH-1-TF	This work
S334	Constitutive P_{hSV40}-driven mammalian expression vector of Gal4-Bcl2 cassette (P_{hSV40}-Gal4-Bcl2-pA), Co-express with S335 to form the split system of CDH-2-TF	This work
S335	Constitutive P_{hSV40}-driven mammalian expression vector of LD3-p65 cassette (P_{hSV40}-LD3-p65-pA)	This work
S336	Constitutive P_{hSV40}-driven mammalian expression vector of Gal4-B-mdm2 cassette (P_{hSV40}-Gal4-mdm2-pA), Co-express with S337 to form the split system of CDH-3-TF	This work
S337	Constitutive P_{hSV40}-driven mammalian expression vector of LD6-p65 cassette (P_{hSV40}-LD6-p65-pA)	This work
S340	Constitutive P_{hSV40}-driven mammalian expression vector of Gal4-CDH-1(Bcl-XL-GS-LD3) cassette (P_{hSV40}-Gal4-CDH-1-pA), Co-express with S341 to form the split system of AIR-1-TF1	[2]

Plasmid	Description and cloning strategy	Reference
S341	Constitutive P_{hSV40}-driven mammalian expression vector of iBcl-XL_v3-p65 cassette (P_{hSV40}-iBcl-XL_v3-p65-pA)	This work
S439	P_{UAS}-driven vector expressing anti-HER2-CAR (P_{5XUAS}-CD8 signal peptide-4D5 anti-HER2-scFv-CD8 hinge-murine 4-1BB-murine CD3z-pA).	This work

Supplementary Table 7: transfection table of CDH and AIRs in cells.

The amount of DNA was calculated per 1 well of a 96-well plate.

The 4D5 scFv-anti HER2-CAR expression assay was performed in 24 well plate, hence, the plasmids of AIR-1-TF for anti-HER2 CAR expression are calculated per well of a 24-well plate.

**Application**	**Reporter**	**Effector**	**Other plasmids**	**Total DNA**
CDH-(1-3)-TF	S132<br>50 ng	S111/S112/S113<br>50 ng	None	100 ng
CDH-(1-3)-TF<br>SV40 promoter	S132<br>50 ng	S133/S134/S135<br>50 ng	None	100 ng
CDH-(1-3)-TF<br>Split system	S132<br>50 ng	S333&S335/S334&S335/S336&S337<br>75 ng/plasmid	None	200 ng
AIR-(1-2)-GEMS	pLS13<br>30ng	S215 + S226<br>/S222 + S228<br>50 ng + 50 ng	**pLS15**<br>**3.3ng**	133.3 ng
AIR-1-TF<br>SEAP reporter	S132<br>50 ng	S340&S341<br>75 ng	None	200 ng
AIR-1-TF<br>Anti-HER2 CAR	S439<br>200ng	S340&S341<br>200 ng	None	600 ng
MIMO-Drug-2+Drug-3	pPKm-118 30 ng<br>pLS13 50 ng	S113 30 ng<br>S215 + S226 50 ng + 50 ng	pLS15 3.3 ng	193.3 ng
SIMO-Drug-1	pPKm-118 30 ng<br>pLS13 50 ng	S111 30 ng<br>S215 + S226 50 ng + 50 ng	pLS15 3.3 ng	193.3 ng
SIMO-Drug-2	pPKm-118 30 ng<br>pLS13 50 ng	S112 30 ng<br>S215 + S226 50 ng + 50 ng	pLS15 3.3 ng	193.3 ng
MISO-Drug-1 + Drug-3	S132 30 ng	S113 30 ng	pLS15 3.3 ng	193.3 ng

Application	Reporter	Effector	Other plasmids	Total DNA
	pLS13 50 ng	S215 + S226 50 ng + 50 ng		
MISO-Drug-2 + Drug-3	S132 30 ng<br>pLS13 50 ng	S113 30 ng<br>S215 + S226 50 ng + 50 ng	pLS15 3.3 ng	193.3 ng

Supplementary Table 8: Summary of tested OFF/ON switches.

Name	Protein components	Disruptor/ Inducer	Corresponsing plasmids	Applications
OFF switches				
CDH-1-TF	Bcl-XL & LD3	Drug-1	S111	OFF switchable TF system
CDH-2-TF	Bcl2 & LD3	Drug-2	S112	OFF switchable TF system
CDH-3-TF	mdm2 & LD6	Drug-3	S113	OFF switchable TF system
CDH-1-GEMS	Bcl-XL & LD3	Drug-1	S184 & S185	OFF switchable GEMS system
Weakened CDH-1-GEMS	Bcl-XL & LD3-v3	Drug-1	S184 & S188	More senstitive OFF switchabe GEMS system
CDH-2-GEMS	Bcl2 & LD3	Drug-2	S193 & S185	OFF switchable GEMS system
Weakened CDH-2-GEMS	Bcl2 & LD3-v3	Drug-2	S193 & S188	More senstitive OFF switchabe GEMS system
CDH-3-GEMS	mdm2 & LD6	Drug-3	S189 & S190	OFF switchable GEMS system
iCDH-1-GEMS	iBcl-XL_v3 & LD3	Drug-1	S226 & S185	insensitive CDH-1
iCDH-2-GEMS	iBcl2_v4 & LD3	Drug-2	S228 & S185	insensitive CDH-2
ON-switches				
AIR-1-GEMS	iBcl-XL_v3 & CDH-1(BclxL-GS-LD3)	Drug-1	S215 & S226	ON switchable GEMS system
AIR-2-GEMS	iBcl2_v4 & CDH-2(Bcl2-GS-LD3)	Drug-2	S222 & S228	ON switchable GEMS system
AIR-1-TF	iBcl-XL_v3 & CDH-1(BclxL-GS-LD3)	Drug-1	S340&S341	ON switchable TF system

Supplementary Table 9: Summary of control logics.

SISO			
CDH-1-TF	Drug-1	SEAP/Luciferase	Fig. 3
CDH-2-TF	Drug-2	SEAP/Luciferase	Fig. 3
CDH-3-TF	Drug-3	SEAP/Luciferase	Fig. 3
CDH-1-GEMS	Drug-1	SEAP	Fig. 3
Weakened CDH-1	Drug-1	SEAP	Fig. 3
CDH-2-GEMS	Drug-2	SEAP	Fig. 3
Weakened CDH-2	Drug-2	SEAP	Fig. 3
CDH-3-GEMS	Drug-3	SEAP	Fig. 3
iCDH-1-GEMS		SEAP	Supp Fig. 8
iCDH-2-GEMS		SEAP	Supp Fig. 9
AIR-1-GEMS	Drug-1	SEAP	Fig. 4
AIR-2-GEMS	Drug-2	SEAP	Fig. 4
AIR-1-TF	Drug-1	SEAP	Fig. 4
MISO			
CDH-1-TF & CDH-3-TF	Drug-1 & Drug-3	SEAP/Luciferase	Fig. 5
CDH-1-GEMS & CDH-3-GEMS	Drug-2 & Drug-3	SEAP	Fig. 5
MIMO			
AIR-1-GEMS & CDH-3-TF	Drug-1 & Drug-3	SEAP + Luciferase	Fig. 5
AIR-2-GEMS & CDH-3-TF	Drug-2 & Drug-3	SEAP + Luciferase	Fig. 5
SIMO			
AIR-1-GEMS & CDH-1-TF	Drug-1	SEAP + Luciferase	Fig. 5
AIR-2-GEMS & CDH-2-TF	Drug-2	SEAP + Luciferase	Fig. 5

References

1. Miller, S., Janin, J., Lesk, A. M. & Chothia, C. Interior and surface of monomeric proteins. *J. Mol. Biol.* **196**, 641–656 (1987).
2. Havranek, J. J. & Harbury, P. B. Automated design of specificity in molecular recognition. *Nat. Struct. Biol.* **10**, 45–52 (2003).
