# Supplementary material for: A rational blueprint for the design of chemically-controlled protein switches
Source: Nat Commun. 2021 Oct 1;12:5754. doi: 10.1038/s41467-021-25735-9 (PMC8486872; doi:10.1038/s41467-021-25735-9)
Supplement: Supplementary file 2 — Reporting Summary [file 41467_2021_25735_MOESM2_ESM.pdf]

nature portfolio

Corresponding author(s): Bruno E. Correia

Last updated by author(s): 1-8-2021

Reporting Summary

Nature Portfolio wishes to improve the reproducibility of the work that we publish. This form provides structure for consistency and transparency in reporting. For further information on Nature Portfolio policies, see our Editorial Policies and the Editorial Policy Checklist.

Statistics

For all statistical analyses, confirm that the following items are present in the figure legend, table legend, main text, or Methods section.

n/a	Confirmed	
☐	☒	The exact sample size (*n*) for each experimental group/condition, given as a discrete number and unit of measurement
☐	☒	A statement on whether measurements were taken from distinct samples or whether the same sample was measured repeatedly
☒	☐	The statistical test(s) used AND whether they are one- or two-sided<br>*Only common tests should be described solely by name; describe more complex techniques in the Methods section.*
☒	☐	A description of all covariates tested
☒	☐	A description of any assumptions or corrections, such as tests of normality and adjustment for multiple comparisons
☐	☒	A full description of the statistical parameters including central tendency (e.g. means) or other basic estimates (e.g. regression coefficient) AND variation (e.g. standard deviation) or associated estimates of uncertainty (e.g. confidence intervals)
☒	☐	For null hypothesis testing, the test statistic (e.g. *F*, *t*, *r*) with confidence intervals, effect sizes, degrees of freedom and *P* value noted<br>*Give P values as exact values whenever suitable.*
☒	☐	For Bayesian analysis, information on the choice of priors and Markov chain Monte Carlo settings
☒	☐	For hierarchical and complex designs, identification of the appropriate level for tests and full reporting of outcomes
☐	☒	Estimates of effect sizes (e.g. Cohen's *d*, Pearson's *r*), indicating how they were calculated

Our web collection on statistics for biologists contains articles on many of the points above.

Software and code

Policy information about availability of computer code

Data collection	Rosetta, Microsoft Excel for Mac (Version 16.51)
Data analysis	Protein design calculation were performed by Rosetta, all scripts are available at: https://github.com/LPDI-EPFL/CDH_AIR.<br>Pymol (The PyMOL Molecular Graphics System, Version 2.0 Schrödinger, LLC) and ChimeraX (UCSF ChimeraX version: 0.94.dev202004140658) were used for structural visualization.<br>Graphpad Prism(Version 8.3.0) used for data analysis.

For manuscripts utilizing custom algorithms or software that are central to the research but not yet described in published literature, software must be made available to editors and reviewers. We strongly encourage code deposition in a community repository (e.g. GitHub). See the Nature Portfolio guidelines for submitting code & software for further information.

Data

Policy information about availability of data

All manuscripts must include a data availability statement. This statement should provide the following information, where applicable:

- Accession codes, unique identifiers, or web links for publicly available datasets
- A description of any restrictions on data availability
- For clinical datasets or third party data, please ensure that the statement adheres to our policy

All data is available in the main text or the supplementary information. The raw data supporting the findings of this study are provided in the source data. Coordinates of the determined structure have been deposited in the protein data bank with accession code PDB id: 7AYE and will be released upon publication.

All code used to perform the design simulation can be found at: https://github.com/LPDI-EPFL/CDH_AIR.
Plasmids encoding the CDH and AIR components available from Addgene(Addgene id: 174549-174553) upon publication.

Field-specific reporting

Please select the one below that is the best fit for your research. If you are not sure, read the appropriate sections before making your selection.

- [x] Life sciences
- [ ] Behavioural & social sciences
- [ ] Ecological, evolutionary & environmental sciences

For a reference copy of the document with all sections, see nature.com/documents/nr-reporting-summary-flat.pdf

Life sciences study design

All studies must disclose on these points even when the disclosure is negative.

Sample size	No sample size calculation was performed. The probability of achieving a reasonable result depends on the true standard error (s) per unit, the number of replication (r), and experimental error (residual) degree of freedom (dfE). Based on the estimation of assay complexity, we decided a n=3 biological replicates for cell culture experiments, which was predicted to be sufficient for estimating the spread of data, for calculating the mean and for detecting statistically significant differences between compared groups. Preliminary results and experience in similar cell culture experiments support n=3 biologically independent samples per individual experiment as sufficient to detect meaningful differences in reporter gene expression.
Data exclusions	We did minimal data exclusion of points where the experiment was technically problematic and the measurements of the reporter proteins yielded negative values. Here is a summary of the excluded data figure 3f (three data points excluded of CDH-2 measurement, 3 out of 42) and figue5i (5 data points of SEAP, 5 out of 42, 4 points of Luciferase, 4 out of 42).
Replication	All cellular experiments were performed with three biological replicates and reported fold changes and EC50s were repeated three times. And all replication attempts were successful.
Randomization	No animal or human research participants were involved. For computational protein design and biochemical characterization, there was no randomization involved. For cell culture experiments, no covariates based on sample allocations to experimental groups could be observed and no randomization was performed. All transfection of drug treated and non-treated, dose-dependent drug treatment were performed with cells transfected under same conditions with the same transfection mix and subsequent addition of drugs. The cells for these experiments were cultured in the same plate with the same well-distribution. For most experiments the inner 60 wells of 96 well plates were used to avoid the effects of evaporation.
Blinding	No animal or human research participants were involved. For cell culture experiments investigators were not blinded. Our workflow makes extensive use of multichannel pipetting for conducting several experiments at the same time. Hence it is unlikely that even subconcious bias regarding anticipated results could influence the data. The parallel conduction of several experiments makes it unlikely that the researcher could identify any given transfection mix, hence elaborate blinding procedures were deemed unnecessary.

Reporting for specific materials, systems and methods

We require information from authors about some types of materials, experimental systems and methods used in many studies. Here, indicate whether each material, system or method listed is relevant to your study. If you are not sure if a list item applies to your research, read the appropriate section before selecting a response.

Materials & experimental systems

n/a	Involved in the study	
[ ]	[x]	Antibodies
[ ]	[x]	Eukaryotic cell lines
[x]	[ ]	Palaeontology and archaeology
[x]	[ ]	Animals and other organisms
[x]	[ ]	Human research participants
[x]	[ ]	Clinical data
[x]	[ ]	Dual use research of concern

Methods

n/a	Involved in the study	
[x]	[ ]	ChIP-seq
[x]	[ ]	Flow cytometry
[x]	[ ]	MRI-based neuroimaging

Antibodies

Antibodies used	biotinylated human Her2/ErbB2 Protein from Acro Biosystems and Avidin-Alexa FLuor PE from Biolegend
Validation	All commercially available antibodies were validated by vendors. Validation statements are provided on the manufacture's website.

Eukaryotic cell lines

Policy information about cell lines

Cell line source(s)	HEK293T cells (ACC635)
Authentication	The cell line was thawed from previously verified cryo stocks and no further authetication was performed.
Mycoplasma contamination	The cell line was not tested for mycoplasma contamination.
Commonly misidentified lines (See ICLAC register)	None
